# Cryptic chytridiomycosis linked to climate and genetic variation in amphibian populations of the southeastern United States

**DOI:** 10.1371/journal.pone.0175843

**Published:** 2017-04-27

**Authors:** Ariel A. Horner, Eric A. Hoffman, Matthew R. Tye, Tyler D. Hether, Anna E. Savage

**Affiliations:** 1 Department of Biology, University of Central Florida, Orlando, Florida, United States of America; 2 Department of Ecology and Genetics, Uppsala University, Uppsala, Sweden; 3 Institute of Ecology and Evolution, University of Oregon, Eugene, Oregon, United States of America; University of Regina, CANADA

## Abstract

North American amphibians have recently been impacted by two major emerging pathogens, the fungus *Batrachochytrium dendrobatidis* (*Bd*) and iridoviruses in the genus *Ranavirus* (*Rv*). Environmental factors and host genetics may play important roles in disease dynamics, but few studies incorporate both of these components into their analyses.

Here, we investigated the role of environmental and genetic factors in driving *Bd* and *Rv* infection prevalence and severity in a biodiversity hot spot, the southeastern United States.

We used quantitative PCR to characterize *Bd* and *Rv* dynamics in natural populations of three amphibian species: *Notophthalmus perstriatus*, *Hyla squirella* and *Pseudacris ornata*. We combined pathogen data, genetic diversity metrics generated from neutral markers, and environmental variables into general linear models to evaluate how these factors impact infectious disease dynamics. Occurrence, prevalence and intensity of *Bd* and *Rv* varied across species and populations, but only one species, *Pseudacris ornata*, harbored high *Bd* intensities in the majority of sampled populations. Genetic diversity and climate variables both predicted *Bd* prevalence, whereas climatic variables alone predicted infection intensity.

We conclude that *Bd* is more abundant in the southeastern United States than previously thought and that genetic and environmental factors are both important for predicting amphibian pathogen dynamics. Incorporating both genetic and environmental information into conservation plans for amphibians is necessary for the development of more effective management strategies to mitigate the impact of emerging infectious diseases.

## Introduction

Infectious disease is a well-known driver of animal declines worldwide (e.g. [1–2]). Ectothermic vertebrates, particularly reptiles and amphibians, have been exceptionally impacted by emerging infectious diseases (EIDs) [1]. Observations of massive, worldwide, pathogen-associated amphibian die-offs date back to the 1970s and 80s [3]. In North America, amphibians have been impacted by two major emerging pathogens, the fungus *Batrachochytrium dendrobatidis* (*Bd*) and a class of iridoviruses collectively known as *Ranavirus* (*Rv*) [4–7]. *Bd* was identified as a disease agent in 1998 [8] and *Rv* was classified as an emerging pathogen after 1993 [9–10]. In North America, *Bd* has mainly impacted anurans in the families Ranidae and Bufonidae, causing mass mortality events for species in these families. Most infections have occurred in the western United States and have impacted already threatened and endangered species (e.g. [1,4, 11–12]). *Rv* has caused die-offs in at least 20 different reptile and amphibian species throughout the United States, including species under state and federal protection [4, 13–14].

In the southeastern United States (hereafter, Southeast), emerging pathogens impacting ectothermic vertebrates have been poorly characterized but have the potential to significantly impact the area. The Southeast is exceptionally rich in amphibian and reptile diversity, hosting more than half of species occurring in the United States [15]. EIDs therefore have potential to severely impact this region’s ecosystems and overall biodiversity. Presently, EIDs have been documented in multiple amphibian and reptile groups occurring in the Southeast [4, 13, 16], yet disease monitoring has been limited. One species, the Gopher frog (*Lithobates capito*), is susceptible to *Rv* infections in the lab [14] and infection and die-offs have been documented for wild populations [16–17]. However, impacts of pathogens on other species in this area are more enigmatic [13, 17–19]. Because disease monitoring has been limited, museum specimens and other archived biological samples are critical for retrospective pathogen detection and can aid in uncovering when pathogens were first introduced and where they were found in the past [20].

Climatic variables are significant drivers of pathogen prevalence in wildlife populations, and amphibians and their pathogens are no exception (e.g. [7, 21–22]). Temperature and precipitation are the two major environmental factors that appear to be drivers of *Bd* dynamics. Studies have shown a negative relationship between temperature and *Bd* occurrence, prevalence and intensity both in the lab [23–24] and in natural populations (e.g. [7, 25–27]). Additionally, variation in precipitation and humidity have been implicated in the occurrence and prevalence of *Bd*, with increased precipitation and humidity driving patterns (e.g. [23, 28]). Most work to date on *Rv* has focused on documenting infections. To our knowledge, there is a paucity of work investigating how environmental factors serve as drivers of *Rv*. Only two studies look at this pattern; one in which salamanders were experimentally infected under different temperature regimes and a negative relationship between *Rv* infection intensity and temperature was found [29] and one testing ranid frogs under differing temperature regimes [30].

There is increasing empirical evidence for a genetic basis to disease resistance in wild vertebrate populations [31–32], although more studies are needed to test this hypothesis in amphibian taxa [33]. To date, reduced genetic diversity has been found to increase susceptibility to one pathogen (*Rv)* in a single amphibian species based on neutral microsatellite loci [34]. Additionally, a handful of studies have explored the underlying genetic basis for disease resistance to *Bd*. Tobler and Schmidt [35] looked at among-population susceptibility to *Bd* in a European frog and inferred that differences in susceptibility among populations had a genetic basis due to differential population responses in a common garden experiment. Similarly, Savage et al. [36] found that neutral genetic diversity was negatively correlated with *Bd* infection prevalence in a North American frog. Immunogenetic analyses have also found significant associations between specific major histocompatibility complex (MHC) class II alleles and *Bd* tolerance in the lab and in natural systems for multiple species of anurans [37–39]. These studies suggest that host genetic diversity underlies differential amphibian population responses to EIDs, but are based on a limited number of taxa.

Despite the importance of environmental and genetic factors in explaining amphibian disease dynamics when investigated separately, few studies incorporate both into a single analysis. Ribas et al. [24] demonstrated in the lab that both temperature and expression of skin peptides determined how anuran hosts responded to *Bd* infection. Savage et al. [39] found that environmental factors were responsible for predicting *Bd* infection intensity, while both genetic and environmental factors influenced *Bd* prevalence. This was the first analysis to combine both genetic and environmental factors in a predictive model for EIDs in a natural amphibian system. More studies are needed to confirm the importance of both genetic and environmental factors for explaining amphibian EIDs.

Here, we assess whether EIDs are impacting amphibian populations in the southeastern United States by characterizing *Bd* and *Rv* dynamics using archived samples from three amphibian species: a salamander, *Notophthalmus perstriatus* and two tree frogs, *Hyla squirella* and *Pseudacris ornata*. Each species exhibits unique life history traits and all three occur throughout the Southeast, offering varying perspectives into pathogen dynamics among diverse taxa. *Notopthalmus perstriatus* has a complex life cycle, spending two out of its three life stages in the water [40]. Previous studies have observed that *N*. *perstriatus* and other *Notophthalmus* are susceptible to both *Bd* [19] and *Rv* [41]. *Hyla squirella* is a common, highly arboreal species that breeds in large aggregates during summer months (Elliot et al. 2009), and little is known about the presence or impact of *Bd* and *Rv* in this species. In contrast, *P*. *ornata* is an increasingly uncommon species (B. Means pers. comm.), breeds in winter at low densities [42], and is highly susceptible to *Rv* in experimental infection trials [43]. Field studies of *P*. *ornata* tadpoles in northern Florida have also confirmed *Rv* infections in the wild [17]. Previous population genetic analyses found that *N*. *perstriatus* form distinct East-West groups that do not share haplotypes [40], *H*. *squirella* genetic structure is heavily determined by habitat structure [44], and *P*. *ornata* genetic structure varies among populations and the species may have been widespread across the Southeast in the past [45]. By combining these genetic data with environmental and disease variables for each sampled population, we simultaneously assessed the importance of host genetics and environmental variables on predicting disease impact and spread in amphibian populations of the Southeast.

## Materials and methods

### Sample collection

All appropriate permits were acquired for the desired field and lab work. Vertebrate animal use was approved by University of Central Florida's IACUC, #06-01W, 09-13W, 09-21W. Samples were collected over various months throughout the Southeast Atlantic Coastal Plain from 1997 to 2010 (Table A in S1 File). *Notophthalmus perstriatus* samples were collected in Florida and Georgia from 1997 to 2000, with additional samples collected in 2008–2010. Samples were collected during various months over the entire collection period [40]. *Hyla squirella* samples were collected in Florida and Georgia in 2010 during summer months [44]. *Pseudacris ornata* samples were collected in Florida, Georgia, Alabama, South Carolina and North Carolina between 2006 and 2009 during winter months [45]. Toe clips were taken from anurans and tail clips were taken from salamanders. Each animal was then released where it was found. For *N*. *perstriatus*, tissue was either stored in saturated salt buffer (NaCl; 25 mM EDTA, pH 7.5; 20% DMSO), or in DrieRite Desiccant [40]. For *H*. *squirella*, tissue was stored in in anhydrous calcium sulfate [44]. *P*. *ornata* tissue samples were also stored in anhydrous calcium sulfate [45]. While multiple storage methods were used, long-term storage occurred in a -20C freezer, we ran a random assemblage of samples (10 samples from each species group) and tested elutions via a Microdrop assay in a BioTek plate reader to check for a comparison of the DNA extractions. No significant difference was found between species in amount of DNA present in elutions (P = .06).

### Pathogen detection

DNA was extracted from whole tissue samples using DNeasy Blood and Tissue kits (Qiagen) or through the phenol—chloroform method [40, 44–45] and DNA elutions were stored at -20 C or cooler. Taqman quantitative (q)PCR was performed on extracted DNA using the Bio-Rad CFX96 Real-Time System and analyzed with Bio-Rad CFX Manager 3.1 software. Reaction volumes were 25 μL for all standards, samples and controls, consisting of: 8 μL of Bio-Rad Super Mix, 2 μL of 10 μM Forward primer (0.8 μM/ μL), 2 μL of 10 μM Reverse primer (0.8 μM/ μL), 3 μL of Molecular Grade water, 5 μL of 1 μM probe (*Bd* or *Rv*; 0.2 μM/ μL) and 5 μL of standard DNA template or sample DNA template. Cycling conditions were as follows: 95°C for 5 minutes followed by 40 cycles of 95°C for 15 seconds and 60°C for 1 min. *Bd* reactions used primers and probes developed by Boyle et al. [46] and *Rv* reactions used primers and probes developed by Allender, Bunick and Mitchell [47]. *Bd* and *Rv* reactions were run separately on individual 96-well plates. For absolute pathogen quantification, standard curves were generated from serial dilutions of synthetic pathogen DNA (gBlock Gene Fragments) run in duplicate [48–49]. Two negative controls (molecular grade water) were included with each run, as well as a positive control. Samples were first run in pools, which consisted of 5 μL of DNA template from each individual within a population combined, to test for the presence of positives within a population. A result was considered positive if the DNA from 5 μL of the pooled sample amplified before cycle 39 for at least two runs. If a population tested positive, each individual was then tested. All positive samples were run twice and the average of the two values was used in downstream analysis. In rare cases when two runs were inconsistent (one positive and one negative, or more than an order of magnitude difference in infection intensity), a third run was performed and the two most consistent results were retained.

### Pathogen data analyses

We used two metrics to catalog *Bd* and *Rv* infection across the species ranges for these three amphibians. Prevalence was calculated by dividing the number of infected individuals by the total population sample size, and 95% Clopper—Pearson binomial confidence intervals were calculated using the package *binom* in R (see S1 File for Data A (R code)). To compare prevalence across population and species, we analyzed two-way contingency tables using Fisher's Exact Tests with simulated *P*-values based on 2000 replicates. The second metric, infection intensity, was calculated as the mean number of Genome Equivalents (GE) among duplicate runs. Mean infection intensity per population was measured as the mean infection intensity among infected individuals only. To compare average infection intensity across populations and species, a two-way ANOVA was performed in R v. 3.1.3 [50]. Prevalence and intensity maps were created in ArcGIS v. 10.2.2 to visualize the spatial distribution of infections.

### Genetic and environmental disease modeling

For species that tested positive for *Bd* or *Rv* in multiple populations, we used general linear models (GLMs) to predict pathogen prevalence (with binomial error; [51]) and the natural log of intensity based on genetic and environmental variables, as well as location. For genetic predictor variables, we used average expected heterozygosity (*H*_E_) and allelic richness (AR) as these were found to be important predictors of infection by Savage et al. [36]. Genetic diversity estimates were calculated by investigating diversity at 7 nuclear microsatellites for *P*. *ornata* [45], as this species was the only one used in modeling due to its infection prevalence recovered (Table B in S1 File). For environmental predictor variables, we used average precipitation, average temperature, and maximum and minimum temperature per population site for each month of the year. Location factors included latitude and longitude for each population site. Environmental data (current data interpolated from 1960–2000 data) was acquired through Worldclim/Bioclim layers in ArcGIS [52]. Environmental and location data were assessed with Principle Component Analysis (PCA). Additive and interactive models were created and assessed via variance inflation factor (vif) using the *car* package in R, and only models with values ≤4 were included [53]. GLMs meeting this criterion were ranked using Akaike information criterion (AICc), and the most informative model was chosen using the lowest AICc value [53].

## Results

**Fig 1:**
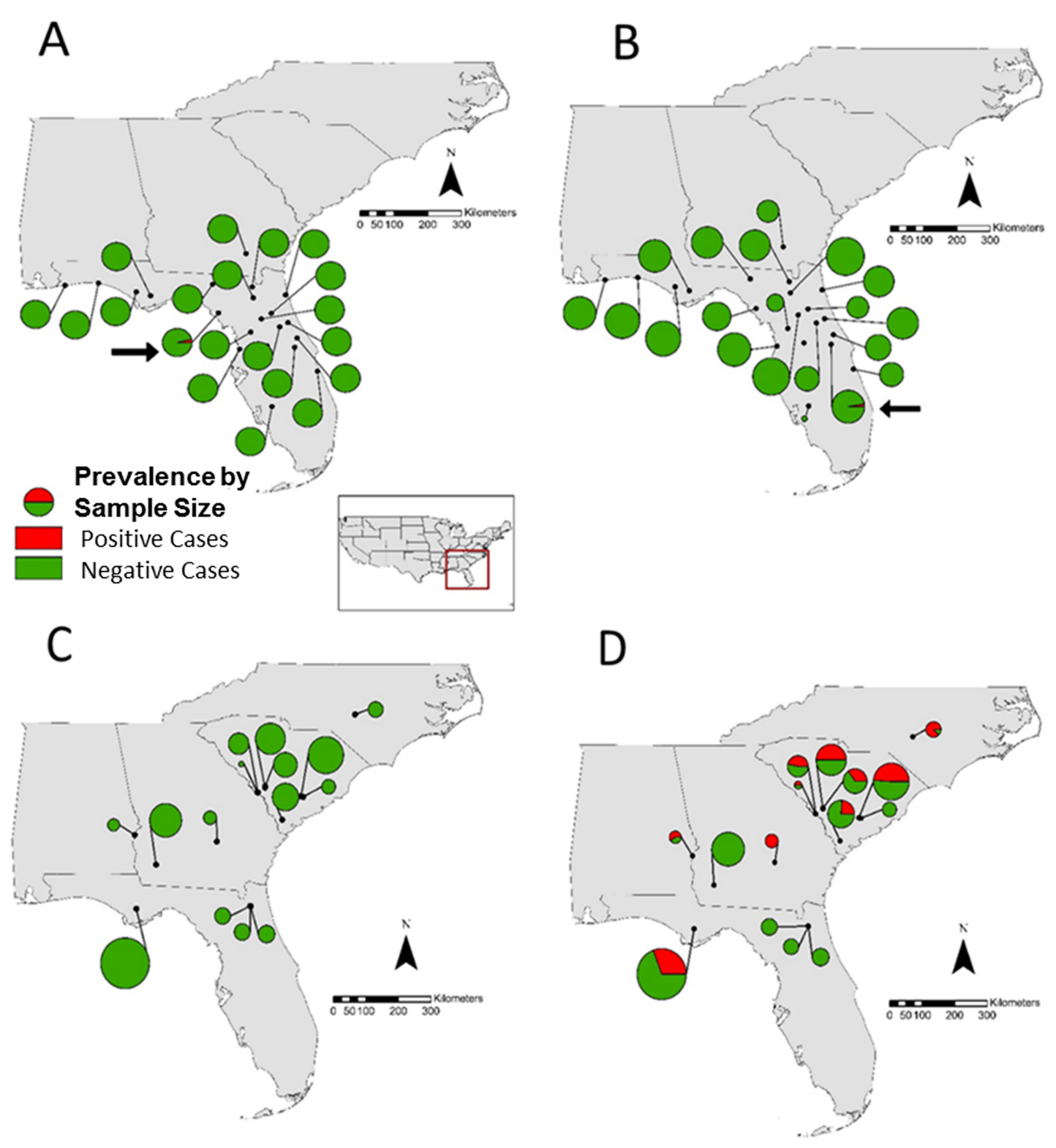
Map of sample localities and (A) *Bd* prevalence in *Hyla squirella*, (B) *Rv* prevalence in *H*. *squirella*, (C) *Bd* prevalence in *Pseudacris ornata* and (D) *Rv* prevalence in *P*. *ornate*. Circle size is relative to population size. Arrows point to infected *H*. *squirella* populations. Green represents proportion of negative cases of the indicated pathogen while red represents proportion of positive cases.

**Fig 2:**
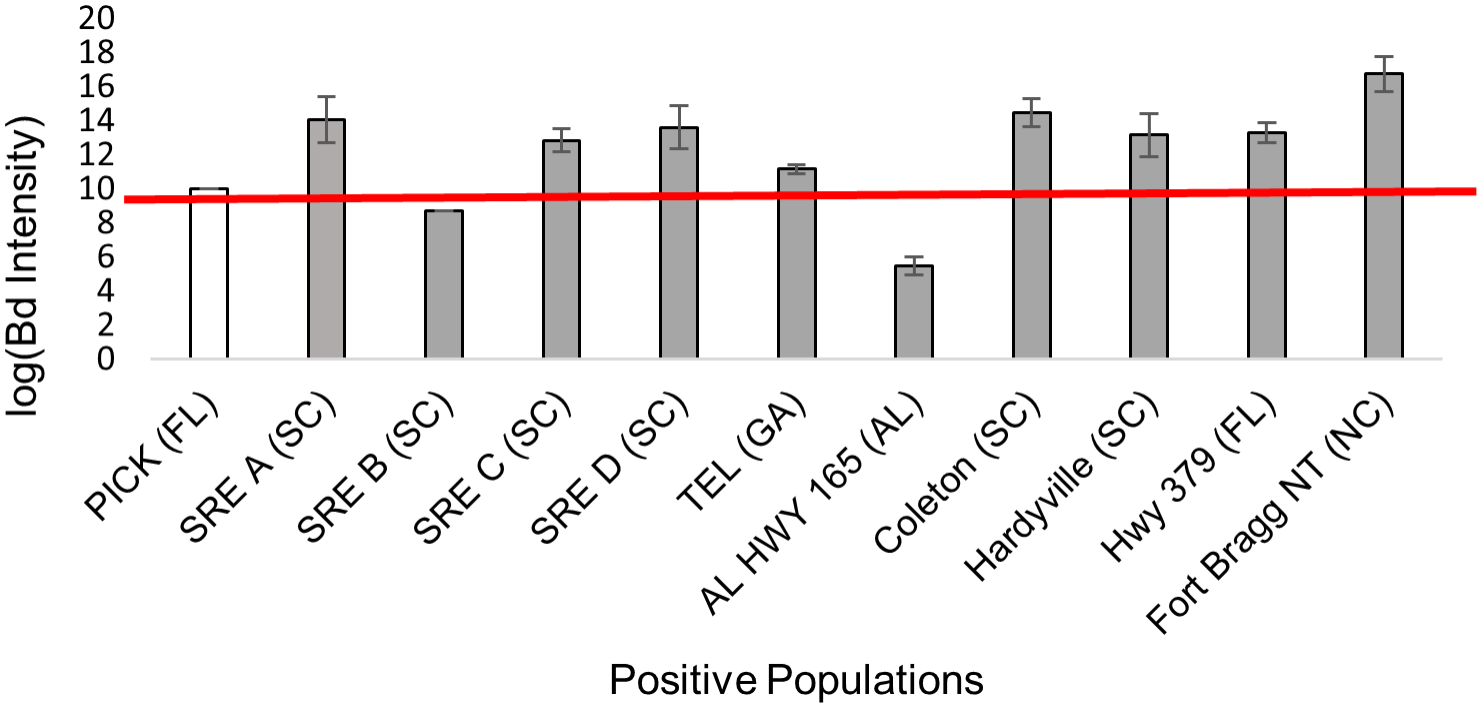
Log-transformed *Bd* average intensities for sampled populations of *H*. *squirella* and *P*. *ornata* with standard error of the mean (SEM). White bars indicate average intensity among infected individuals from *H*. *squirella* populations and black bars denote average intensity among infected individuals from *P*. *ornata* populations. The red line marks an infection intensity of 10,000 GE.

We tested 401 *N*. *perstriatus* tissue samples from 11 populations, none of which were infected with *Bd* or *Rv*. Among 580 *H*. *squirella* tissue samples from 20 populations, one individual was infected with *Bd* (Table C in S1 File; Fig 1a) and one individual from a different population tested positive for *Rv* (Table C in S1 File; Fig 1b). Neither *Bd* nor *Rv* prevalence were significantly different among *H*. *squirella* populations (Fisher Exact test *P* = 0.64). Finally, among 327 *P*. *ornata* tissue samples from 15 populations, 103 individuals from 10 populations were *Bd* positive (Table A in S1 File; Fig 1c), whereas none tested positive for *Rv* (Table C in S1 File; Fig 1d). *Bd* prevalence among infected *P*. *ornata* populations ranged from 0.24 to 1.0 (Table C in S1 File). *Bd* prevalence varied significantly among *P*. *ornata* populations (Fisher Exact test, *P* = 0.0005). Additionally, pooled *Bd* prevalence was significantly different in *P*. *ornata* compared to *H*. *squirella* (Fisher Exact test, *P* < 0.00001). Average *Bd* infection intensity among infected populations ranged from 226 to almost eighteen million genome equivalents (GE; Fig 2). Of the 11 infected populations, nine harbored average infections of over 10,000 GE (Table C in S1 File; Fig 2). All but one infected populations had 40% of individuals harboring loads above 10,000 GE and two populations had 100% of individuals with infections above 10,000 GE. *Bd* infection intensity was significantly different among infected *P*. *ornata* populations (two-way ANOVA, *P* < 0.00001).

**Fig 3:**
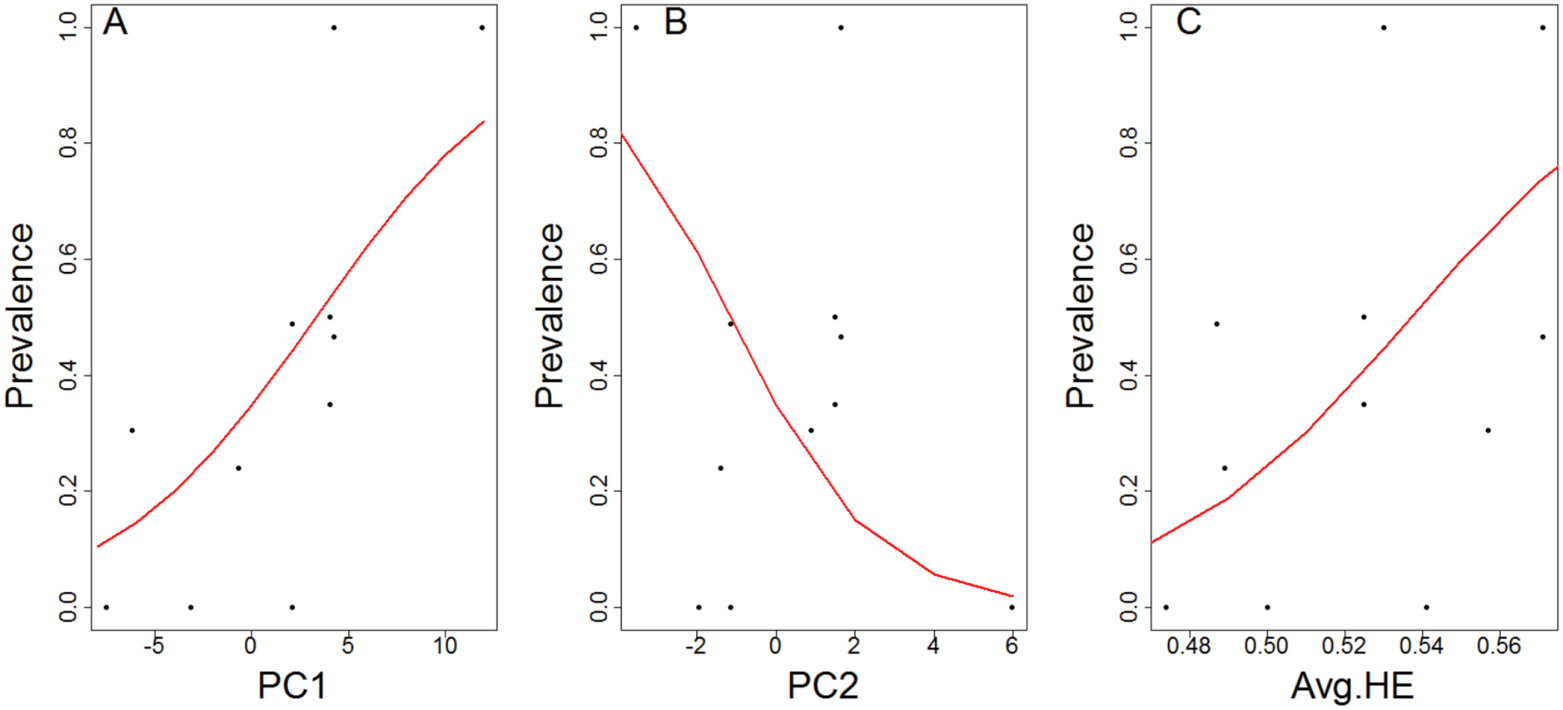
Logistic relationships between *Bd* prevalence and PC1 (A), PC2 (B), and average heterozygosity (C) in populations of *P*. *ornata*. Increasing values of PC1 correspond most strongly to decreasing values of temperature. Increasing values of PC2 correspond most strongly to increasing winter precipitation. In all panels, each dot corresponds to the observed prevalence in a population and each line corresponds to the best fit logistic model of the relationship between the two variables shown.

Because *P*. *ornata* was the only species harboring pathogens in more than one population, and *Bd* was the only pathogen detected, we limited GLM analyses to *Bd* dynamics within *P*. *ornata*. PCA of environmental variables across sampled *P*. *ornata* populations revealed that the first two components explained 89% of variation in our data (Fig A in S1 File). PC1 is associated with decreasing temperature and some increasing precipitation variables and positively associated with latitude. PC2 is positively associated with winter precipitation. We assessed only additive models with AICs for prevalence and intensity as interactive models showed high variance inflation. We chose the most informative models based on the lowest AIC score in the set (Table 1). PC1, PC2 and average heterozygosity significantly influenced *Bd* prevalence (Fig 3; Table D in S1 File). Based on our general linear models, there is a significant relationship between decreasing temperature and an increase in *Bd* prevalence (*P* = 0.0000002; Fig 3A). Moreover, there is a significant negative relationship between winter precipitation and *Bd* prevalence (*P* = 0.000591; Fig 3B). Surprisingly, our model identified a significant relationship between increased average heterozygosity and increased *Bd* prevalence (*P* = 0.0000698; Fig 3C). Environmental factors (PC1) significantly influenced *Bd* intensity (Fig B and Table E in S1 File). Linear regression displayed a significant relationship between a decrease in temperature and an increase of *Bd* intensity (*P* = 0.011; Fig B in S1 File).

**Table 1:** Five most informative general linear models and the null models for *Bd* prevalence and *Bd* intensity.

***Bd* Prevalence models**	**AICc**	**dAICc**	**Df**	**Weight**
PC1+PC2+AvgHE	62.4	0	4	0.750
PC1+PC2+AvgHE+AR	64.6	2.2	5	0.249
PC1+PC2+AR	75.6	13.2	3	0.001
PC1+PC2	80.4	18.0	2	<0.001
PC1+AvgHE	82.6	20.3	3	<0.001
NULL	112.6	50.3	1	<0.001
***Bd* Intensity models**	**AICc**	**dAICc**	**Df**	**Weight**
PC1	87.0	0	3	0.5192
PC1+AR	89.6	2.6	4	0.1425
PC1+AvgHE	90.7	3.7	4	0.0823
PC1+PC2	91.3	4.3	4	0.0601
AvgHE	91.8	4.8	3	0.0478
NULL	91.5	4.5	2	0.0559

AvgHE = average heterozygosity, AR = allelic richness, dAICc = delta Akaike information criterion. The difference in AICc between the current model and the most informative model in the set. df = degrees of freedom.

## Discussion

Our study used both environmental and genetic variables to create a predictive model for chytridiomycosis disease dynamics in the southeastern United States. We identified both concordant and discordant patterns of pathogen prevalence and infection intensity in *N*. *perstriatus*, *H*. *squirella*, and *P*. *ornata* compared to previous studies [18–19, 54]. First, our data showed limited *Rv* occurrence and high variation in *Bd* infection prevalence within and among our three focal species. Second, we found surprisingly high *Bd* infection intensity in *P*. *ornata* and *H*. *squirella*, a strikingly different result compared to the extremely low intensities previously detected [54]. Finally, our overall model found that both genetic and environmental variables predict *Bd* prevalence but only environmental variables predict infection intensity. These results are important for understanding the enigmatic story of *Bd* infection in this region, and provide a framework for future management of declining amphibians in the Southeast. Overall, this study offers insight into pathogen infection history and amphibian disease dynamics in the southeastern United States, a hotspot of amphibian diversity.

Our three focal amphibian species showed largely unique patterns of *Bd* and *Rv* infection prevalence compared to previous studies on the same or similar species. Interestingly, *Rv* was previously found within ponds where *N*. *perstriatus* populations occur [17], and a closely related *Notophtahlmus* species in the Southeast, *N*. *viridescens*, was found to have high *Bd* infection prevalence within sampled populations [19]. In contrast, we found no evidence of *Bd* or *Rv* in *N*. *perstriatus*, suggesting the presence of genetic, temporal or seasonal differences for our sampled populations. *Hyla squirella* infection dynamics were also surprising as this species breeds in large aggregates in habitats frequented by known *Bd* and *Rv* vector species, particularly *Lithobates catesbianus* [42, 55]. We predicted high pathogen prevalence in *H*. *squirella*, but instead only found two infected individuals (one *Bd* infected and one *Rv* infected) among all sampled populations. Interestingly, these are the first documented *Bd* and *Rv* infections in *H*. *squirella* despite previous sampling efforts [19]. These results indicate low overall infection prevalence in *H*. *squirella*, but the high *Bd* infection intensity we detected in one individual suggests the potential for negative impacts on populations where infection does occur. The only focal species with high pathogen prevalence was *P*. *ornata*, which exhibited strikingly different infection patterns for *Rv* and *Bd* compared to *H*. *squirella*. While *Rv* infection was undetectable in *P*. *ornata*, high *Bd* infection prevalence and intensity occurred in the majority of sampled populations. These results mirror previous studies testing *P*. *ornata* and other members of *Pseudacris* for pathogens in the Southeast [19, 54].

Life history variation among amphibian species often contributes to variation in pathogen infection prevalence [56]. The distinct life histories among our three study species may be an underlying factor contributing to the significant differences in *Bd* prevalence and intensity that we observed. In particular, high susceptibility to *Bd* infection in *P*. *ornata* may be due to the trait of breeding exclusively during rainy periods in the winter months [42], unlike the other two species that are summer breeders. Our data, along with data from several other studies, correlate *Bd* infection with cooler temperatures and higher precipitation in winter months [7, 26–27, 56–57]. Thus, high contact rates among *P*. *ornata* individuals during winter breeding aggregations when *Bd* experiences preferred temperatures may be driving the observed high infection prevalence and intensity. Our results, combined with previous monitoring efforts, serve as a valuable baseline should infection outbreaks occur for other southeastern amphibian species and may help elucidate reasons for any enigmatic declines.

Our *Bd* infection intensity data seem to contradict previous studies suggesting that values greater than 10,000 GE lead to mortality, regardless of the amphibian species [58–59]. We found high *Bd* intensities that surpassed this threshold for the single infected *H*. *squirella* individual and within most infected *P*. *ornata* individuals (Fig 2). Although high *Bd* infection prevalence in *Pseudacris* populations is well documented in the literature [19–20, 54], only one other study quantified *Bd* intensity for *P*. *ornata*; they found low values (<102 GE) for all individuals sampled throughout the United States [54]. We uncovered a pattern that is much more extreme; average *Bd* intensities were millions of GE for all but three infected populations (Fig 2) despite the absence of any observed disease signs or mortality events (T. Hether pers. comm.). Our data therefore demonstrate that the 10,000 GE proposed mortality threshold is not a standard applicable to every species. Indeed, our findings reinforce recent *Bd* studies in Brazilian amphibian communities [60] and New York State amphibian communities [61] which showed varying infection intensities across a range of species and seasons without observing any mortality or disease signs in the individuals sampled.

To uncover the driving forces behind the observed patterns of *Bd* infection among our sampled *P*. *ornata* populations, we incorporated climatic variables and genetic diversity factors into a comprehensive model. *Bd* intensity increased at lower air temperatures, consistent with similar analyses in other species and regions (e.g. [27, 36, 56, 62]. Further, the same variables that influenced *Bd* prevalence for *P*. *ornata* in our study (temperature, precipitation and average heterozygosity) also explained *Bd* prevalence in *L*. *yavapaiensis* in Arizona [36]. However, in contrast to the negative correlation between average heterozygosity and *Bd* prevalence found for *L*. *yavapaiensis*, we found average heterozygosity was positively correlated with *Bd* prevalence for *P*. *ornata* (Fig 3C). This pattern is in direct contrast with expectations, as numerous studies across wildlife disease systems have found higher genetic diversity within populations leads to decreased infection prevalence and increased disease resistance (e.g. [34, 63–64]. Our models show that for *P*. *ornata*, the opposite is true: increased genetic diversity correlates positively with *Bd* prevalence. Two possible explanations exist for this pattern. First, because average heterozygosity increases with larger effective population size, there could be better facilitation of pathogen spread due to density-dependent disease outbreaks [65–66]. This explanation is unlikely for *P*. *ornata*, however, as this species is generally uncommon and has historically small population sizes [42]. Another possible explanation is that *Bd* swept through *P*. *ornata* populations before our sampling occurred, and selection favoring *Bd* tolerant individuals was strong enough to push tolerant genotypes towards fixation, resulting in decreased heterozygosity. *Bd* has been present in the United States long enough to make this “genetic purging” [67] scenario plausible; Ouelett et al. [20] and Talley et al. [68] found evidence of *Bd* infections existing in North America as far back as the late 1800s. Our results could thus represent indirect evidence of genetic tolerance to *Bd* evolving in natural amphibian populations [39], although further genetic sampling, molecular tests of selection and experimental evidence of *Bd* tolerance are necessary to resolve this hypothesis.

Our data strongly suggest that both genetic and environmental factors should be incorporated, when possible, into models when trying to predict dynamics of infectious pathogens in natural populations. Management plans often only consider genetic or environmental factors when planning for long-term species persistence, but it is becoming increasingly clear that both are important for predicting pathogen impacts. While our study only focuses on amphibians, this modeling framework is applicable and important for other wildlife disease systems. Our results also suggest that *Bd* may be more of a concern for the Southeast than previously thought, at least for some species. There are no documented instances of disease-driven morbidity or mortality in *P*. *ornata*, yet a majority of sampled individuals were heavily infected with *Bd* and there is evidence of population declines in recent decades (B. Means pers.comm.). Cryptic chytridiomycosis may therefore be an unobserved but causal factor behind population declines and patterns of genetic diversity. Alternately, the *Bd* strain(s) present in the Southeast may currently exist as commensals or sub-lethal pathogens in *P*. *ornata*. Even under the latter scenario, monitoring *Bd* and other pathogen dynamics is important for future *P*. *ornata* conservation efforts. If novel biotic or abiotic stressors appear, the additional toll of harboring massive *Bd* intensities may be a tipping point towards extirpation. This may be especially true for populations with low genetic diversity, even if that loss of diversity is due to selection for pathogen tolerance. Means and Means (unpublished data) highlight that habitat destruction and degradation are threatening *P*. *ornata* population persistence, and more recently Means et al. [17] found wild *P*. *ornata* tadpoles to be heavily infected with *Rv*. Our results suggest *Bd* is a threat for adult frogs, particularly if the same populations are impacted by *Rv* prior to metamorphosis.

Overall, our study highlights how species are differentially impacted by EIDs in the Southeast and how models can be used to infer which environmental and genetic factors are drivers of infection. Infectious disease is often implicated in amphibian population declines only after morbidity and mortality is observed, making the trigger for a disease outbreak difficult to determine retrospectively. It has therefore become increasingly important to characterize and monitor species that have yet to display signs of disease in order to generate a baseline of pathogen dynamics should any future disease outbreak occur. Museum collections and specimens collected for non-disease studies are invaluable for assessing conditions faced by amphibians in the past [20], and here we utilized these resources to document the presence of two infectious pathogens in two frog species without any prior evidence of disease. Whether ubiquitous *Bd* infections in *P*. *ornata* reflect post-epidemic adaptation, non-pathogenic *Bd* strains, or virulent, ongoing chytridiomycosis that has gone undetected will require additional analyses. Regardless, our modeling results highlight the combined importance of host genetic variation and climate for determining *Bd* prevalence. Other climatic factors, such as seasonality, may also play a big part in disease dynamics and should be considered in future studies. Our results begin the journey to uncovering amphibian pathogen dynamics and can be used to develop more robust predictive models to assess where pathogens will likely spread and to inform species managers, as well as target suitable future re-introduction sites for amphibians that have been hit the hardest by disease-related declines.

## Supporting information

S1 File Contains Tables A-E, Figures A-B, and Data A (R code). (DOCX)
