# Correction: Cryptic chytridiomycosis linked to climate and genetic variation in amphibian populations of the southeastern United States

**DOI:** 10.1371/journal.pone.0186066

**Published:** 2017-10-03

**Authors:** Ariel A. Horner, Eric A. Hoffman, Matthew R. Tye, Tyler D. Hether, Anna E. Savage

There is an error in the first sentence of the “Genetic and environmental disease modeling” subsection of the Methods section. The correct sentence is: For species that tested positive for *Bd* or *Rv* in multiple populations, we used general linear models (GLMs) weighted based on population size to predict pathogen prevalence (with binomial error; [51]) and the natural log of intensity based on genetic and environmental variables, as well as location.

There is an error in Fig 1. Panels C and D appear in the incorrect order and the caption contains spelling errors. Please see the corrected Fig 1 and its caption here.

**Fig 1:**
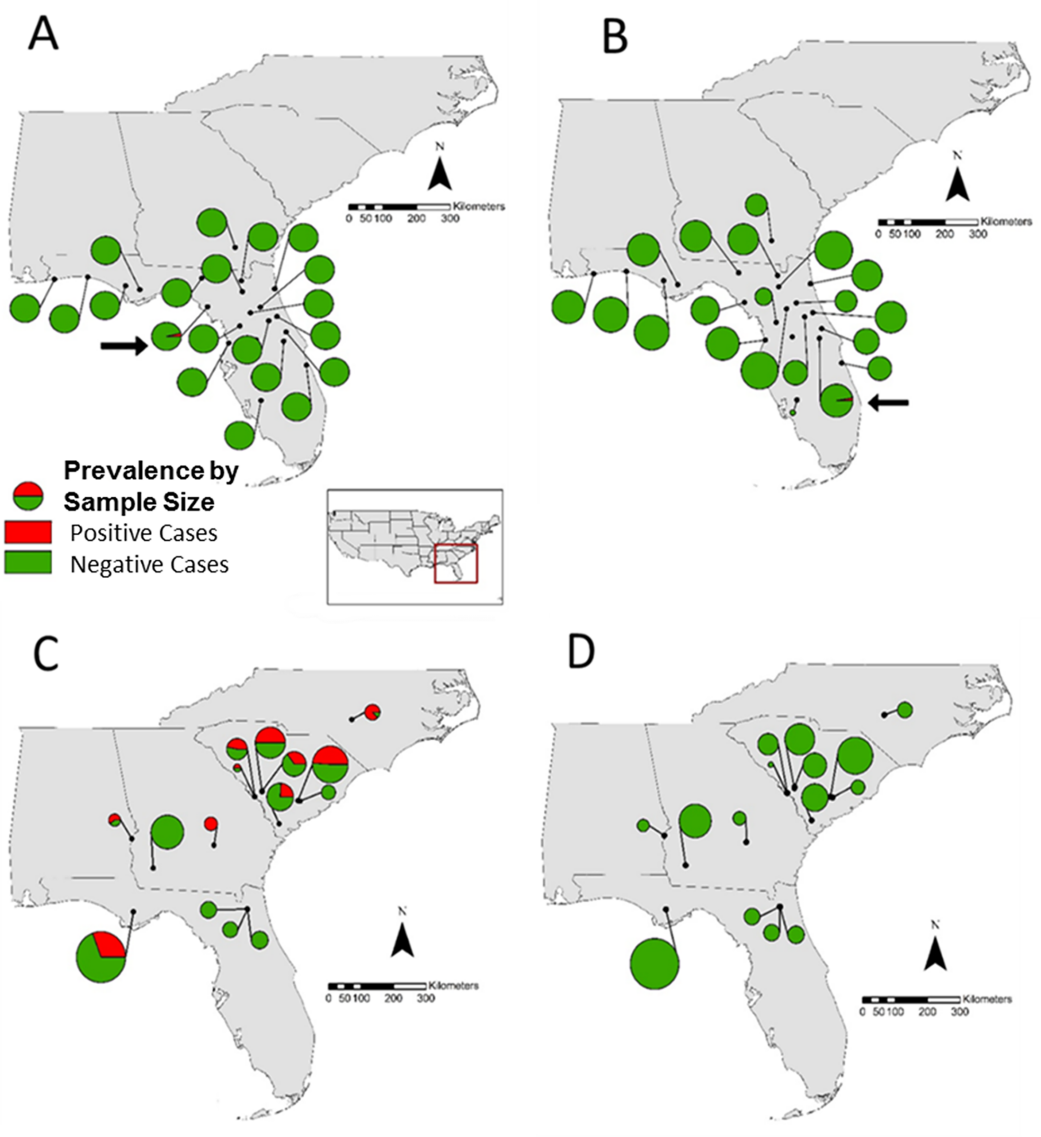
Map of sample localities and (A) *Bd* prevalence in *Hyla squirella*, (B) *Rv* prevalence in *H*. *squirella*, (C) *Bd* prevalence in *Pseudacris ornata* and (D) *Rv* prevalence in *P*. *ornata*. Circle size is proportional to sample size. Arrows point to infected *H*. *squirella* populations. Green represents proportion of negative cases of the indicated pathogen while red represents proportion of positive cases.
