# Supplementary material for: Cryptic chytridiomycosis linked to climate and genetic variation in amphibian populations of the southeastern United States
Source: PLoS One. 2017 Apr 27;12(4):e0175843. doi: 10.1371/journal.pone.0175843 (PMC5407605; doi:10.1371/journal.pone.0175843)
Supplement: S1 File — (DOCX) [file pone.0175843.s001.docx]

**Electronic Supplementary Material**

Cryptic chytridiomycosis linked to climate and genetic variation in amphibian populations of the southeastern United States

Ariel A. Horner, Eric A. Hoffman, Matthew R. Tye, Tyler D. Hether, and Anna E. Savage

Table A. Raw data for Pseudacris ornata genetic loci.

| Pop | Pop Code | PCRU09 | | PCRU14 | | PCRU24 | | POR105 | | PTRI29 | | POR165 | | PCRU10 | |
| --- | --- | --- | --- | --- | --- | --- | --- | --- | --- | --- | --- | --- | --- | --- | --- |
| JEN | JEN001 | 147 | 147 | 181 | 181 | 263 | 263 | 0 | 0 | 162 | 162 | 0 | 0 | 0 | 0 |
|  | JEN002 | 147 | 147 | 181 | 183 | 263 | 265 | 0 | 0 | 162 | 162 | 254 | 268 | 405 | 451 |
|  | JEN003 | 141 | 147 | 0 | 0 | 251 | 266 | 270 | 270 | 162 | 162 | 268 | 0 | 449 | 449 |
|  | JEN004 | 141 | 145 | 181 | 181 | 263 | 271 | 0 | 0 | 162 | 162 | 226 | 0 | 449 | 449 |
|  | JEN005 | 141 | 147 | 181 | 181 | 263 | 263 | 292 | 0 | 162 | 162 | 254 | 268 | 449 | 449 |
|  | JEN006 | 141 | 143 | 181 | 181 | 263 | 266 | 272 | 272 | 162 | 162 | 222 | 300 | 449 | 449 |
|  | JEN007 | 141 | 143 | 181 | 181 | 263 | 0 | 272 | 272 | 162 | 162 | 234 | 256 | 449 | 479 |
|  | JEN008 | 143 | 145 | 181 | 183 | 263 | 263 | 254 | 272 | 162 | 162 | 0 | 0 | 449 | 449 |
|  | JEN009 | 143 | 147 | 181 | 181 | 263 | 263 | 272 | 0 | 162 | 162 | 234 | 258 | 383 | 451 |
|  | JEN010 | 141 | 141 | 181 | 181 | 263 | 272 | 270 | 270 | 162 | 162 | 0 | 0 | 449 | 449 |
|  | JEN011 | 141 | 141 | 181 | 181 | 263 | 263 | 272 | 272 | 162 | 162 | 232 | 258 | 449 | 489 |
|  | JEN012 | 143 | 147 | 183 | 183 | 0 | 0 | 272 | 274 | 162 | 162 | 0 | 0 | 449 | 449 |
|  | JEN013 | 143 | 147 | 181 | 181 | 263 | 263 | 272 | 272 | 162 | 162 | 248 | 0 | 407 | 449 |
|  | JEN014 | 141 | 143 | 181 | 181 | 263 | 263 | 272 | 272 | 162 | 162 | 0 | 0 | 449 | 449 |
|  | JEN015 | 141 | 141 | 181 | 181 | 263 | 263 | 230 | 0 | 162 | 162 | 224 | 0 | 449 | 449 |
|  | JEN016 | 143 | 147 | 181 | 181 | 263 | 263 | 274 | 274 | 162 | 162 | 234 | 234 | 449 | 449 |
|  | JEN017 | 143 | 147 | 181 | 181 | 261 | 265 | 272 | 272 | 0 | 0 | 254 | 268 | 0 | 0 |
|  | JEN018 | 147 | 147 | 181 | 181 | 263 | 263 | 278 | 278 | 162 | 162 | 224 | 0 | 449 | 449 |
|  | JEN019 | 141 | 141 | 181 | 181 | 263 | 263 | 286 | 0 | 162 | 162 | 224 | 232 | 383 | 449 |
|  | JEN020 | 141 | 141 | 181 | 181 | 263 | 263 | 288 | 0 | 162 | 162 | 0 | 0 | 383 | 449 |
|  | JEN021 | 143 | 143 | 181 | 181 | 263 | 263 | 270 | 270 | 162 | 162 | 248 | 254 | 449 | 449 |
|  | JEN022 | 143 | 147 | 181 | 181 | 263 | 263 | 272 | 0 | 162 | 162 | 0 | 0 | 449 | 449 |
|  | JEN023 | 141 | 143 | 181 | 181 | 263 | 263 | 292 | 0 | 162 | 162 | 254 | 254 | 449 | 449 |
|  | JEN024 | 143 | 147 | 181 | 183 | 263 | 265 | 274 | 288 | 162 | 162 | 224 | 0 | 383 | 449 |
|  | JEN025 | 143 | 147 | 183 | 183 | 263 | 0 | 262 | 270 | 162 | 162 | 232 | 232 | 383 | 449 |
|  | JEN026 | 147 | 147 | 181 | 181 | 263 | 263 | 288 | 0 | 162 | 162 | 248 | 254 | 449 | 449 |
|  | JEN027 | 143 | 147 | 181 | 181 | 0 | 0 | 270 | 274 | 162 | 162 | 232 | 248 | 489 | 0 |
|  | JEN028 | 147 | 147 | 181 | 181 | 0 | 0 | 0 | 0 | 162 | 162 | 232 | 232 | 449 | 449 |
|  | JEN029 | 143 | 147 | 181 | 181 | 263 | 263 | 288 | 0 | 162 | 162 | 226 | 232 | 383 | 383 |
| SRE A-D | SRE001 | 143 | 143 | 183 | 183 | 257 | 263 | 226 | 254 | 162 | 162 | 274 | 0 | 449 | 449 |
|  | SRE002 | 141 | 153 | 183 | 183 | 263 | 263 | 230 | 306 | 162 | 162 | 232 | 258 | 449 | 449 |
|  | SRE003 | 143 | 145 | 183 | 183 | 263 | 263 | 270 | 270 | 162 | 162 | 254 | 0 | 0 | 0 |
|  | SRE056 | 141 | 141 | 183 | 183 | 0 | 0 | 0 | 0 | 162 | 162 | 0 | 0 | 0 | 0 |
|  | SRE057 | 141 | 143 | 179 | 181 | 263 | 263 | 162 | 306 | 154 | 162 | 240 | 240 | 449 | 449 |
|  | SRE058 | 141 | 143 | 181 | 183 | 263 | 263 | 162 | 306 | 154 | 162 | 220 | 220 | 0 | 0 |
|  | SRE059 | 143 | 143 | 181 | 183 | 263 | 263 | 158 | 226 | 162 | 162 | 220 | 258 | 449 | 449 |
|  | SRE060 | 143 | 143 | 181 | 183 | 257 | 263 | 274 | 278 | 154 | 162 | 220 | 294 | 443 | 443 |
|  | SRE061 | 143 | 153 | 183 | 183 | 263 | 265 | 162 | 0 | 162 | 162 | 220 | 232 | 449 | 449 |
|  | SRE062 | 143 | 145 | 181 | 183 | 263 | 265 | 254 | 262 | 162 | 162 | 250 | 0 | 449 | 449 |
|  | SRE063 | 143 | 143 | 181 | 183 | 257 | 263 | 246 | 266 | 154 | 154 | 224 | 258 | 443 | 449 |
|  | SRE064 | 141 | 143 | 181 | 183 | 0 | 0 | 226 | 230 | 162 | 162 | 258 | 258 | 449 | 449 |
|  | SRE065 | 143 | 143 | 179 | 181 | 257 | 263 | 174 | 258 | 154 | 162 | 246 | 0 | 443 | 443 |
|  | SRE066 | 143 | 145 | 183 | 183 | 263 | 263 | 230 | 266 | 162 | 162 | 248 | 248 | 0 | 0 |
|  | SRE067 | 143 | 143 | 183 | 183 | 263 | 263 | 226 | 250 | 162 | 162 | 206 | 248 | 0 | 0 |
|  | SRE068 | 143 | 145 | 183 | 183 | 263 | 263 | 162 | 230 | 162 | 162 | 246 | 246 | 0 | 0 |
|  | SRE004 | 143 | 143 | 183 | 183 | 263 | 263 | 162 | 262 | 162 | 162 | 240 | 274 | 441 | 443 |
|  | SRE005 | 143 | 145 | 183 | 183 | 263 | 263 | 258 | 282 | 162 | 162 | 0 | 0 | 449 | 449 |
|  | SRE006 | 143 | 145 | 179 | 183 | 263 | 263 | 0 | 0 | 162 | 162 | 244 | 268 | 449 | 449 |
|  | SRE007 | 143 | 143 | 173 | 183 | 263 | 263 | 226 | 278 | 162 | 162 | 236 | 244 | 443 | 449 |
|  | SRE008 | 143 | 143 | 183 | 183 | 263 | 263 | 222 | 282 | 162 | 162 | 254 | 0 | 449 | 449 |
|  | SRE009 | 141 | 143 | 181 | 183 | 263 | 263 | 222 | 226 | 154 | 162 | 244 | 278 | 383 | 449 |
|  | SRE010 | 141 | 143 | 183 | 183 | 263 | 263 | 246 | 258 | 154 | 162 | 244 | 244 | 443 | 449 |
|  | SRE011 | 143 | 153 | 0 | 0 | 263 | 263 | 262 | 274 | 162 | 162 | 252 | 280 | 443 | 449 |
|  | SRE012 | 141 | 143 | 183 | 183 | 263 | 263 | 230 | 230 | 154 | 162 | 248 | 248 | 449 | 449 |
|  | SRE013 | 143 | 143 | 181 | 183 | 263 | 0 | 234 | 0 | 162 | 162 | 206 | 244 | 449 | 449 |
|  | SRE014 | 143 | 145 | 183 | 183 | 0 | 0 | 254 | 278 | 162 | 162 | 0 | 0 | 443 | 449 |
|  | SRE015 | 143 | 145 | 183 | 183 | 263 | 263 | 162 | 258 | 154 | 162 | 248 | 248 | 449 | 449 |
|  | SRE016 | 143 | 145 | 183 | 183 | 263 | 263 | 174 | 204 | 162 | 162 | 244 | 248 | 443 | 449 |
|  | SRE017 | 141 | 143 | 181 | 183 | 263 | 263 | 182 | 282 | 162 | 162 | 0 | 0 | 425 | 451 |
|  | SRE018 | 143 | 143 | 179 | 183 | 263 | 263 | 230 | 254 | 162 | 162 | 206 | 248 | 443 | 449 |
|  | SRE019 | 145 | 145 | 181 | 183 | 263 | 263 | 222 | 258 | 162 | 162 | 218 | 256 | 443 | 449 |
|  | SRE020 | 141 | 143 | 183 | 183 | 263 | 263 | 230 | 274 | 162 | 162 | 228 | 252 | 0 | 0 |
|  | SRE021 | 143 | 143 | 181 | 181 | 263 | 263 | 250 | 262 | 154 | 162 | 238 | 238 | 443 | 443 |
|  | SRE022 | 143 | 153 | 183 | 183 | 263 | 0 | 258 | 286 | 162 | 162 | 250 | 256 | 449 | 449 |
|  | SRE023 | 143 | 143 | 183 | 183 | 263 | 263 | 230 | 262 | 154 | 162 | 240 | 250 | 443 | 451 |
|  | SRE024 | 141 | 143 | 183 | 183 | 263 | 263 | 254 | 278 | 158 | 162 | 222 | 246 | 443 | 443 |
|  | SRE025 | 141 | 143 | 183 | 183 | 263 | 269 | 226 | 250 | 162 | 162 | 244 | 244 | 443 | 449 |
|  | SRE026 | 143 | 145 | 179 | 183 | 263 | 263 | 266 | 266 | 162 | 162 | 250 | 256 | 449 | 449 |
|  | SRE027 | 143 | 151 | 181 | 183 | 263 | 263 | 226 | 258 | 162 | 162 | 288 | 0 | 0 | 0 |
|  | SRE028 | 143 | 143 | 181 | 183 | 263 | 263 | 162 | 278 | 162 | 162 | 226 | 248 | 449 | 449 |
|  | SRE029 | 143 | 143 | 181 | 183 | 263 | 263 | 222 | 282 | 154 | 162 | 228 | 232 | 443 | 449 |
|  | SRE030 | 143 | 143 | 181 | 181 | 263 | 269 | 246 | 266 | 162 | 162 | 0 | 0 | 383 | 443 |
|  | SRE031 | 143 | 155 | 183 | 183 | 263 | 263 | 258 | 262 | 154 | 154 | 230 | 0 | 449 | 449 |
|  | SRE032 | 145 | 153 | 183 | 183 | 263 | 263 | 226 | 258 | 162 | 162 | 244 | 250 | 383 | 443 |
|  | SRE033 | 143 | 143 | 181 | 183 | 263 | 263 | 254 | 278 | 162 | 162 | 244 | 244 | 443 | 443 |
|  | SRE034 | 141 | 143 | 181 | 183 | 263 | 263 | 254 | 282 | 162 | 162 | 220 | 244 | 449 | 449 |
|  | SRE035 | 143 | 145 | 181 | 181 | 263 | 263 | 262 | 282 | 162 | 162 | 234 | 252 | 443 | 451 |
|  | SRE036 | 143 | 143 | 181 | 183 | 263 | 263 | 222 | 254 | 162 | 162 | 206 | 252 | 451 | 451 |
|  | SRE037 | 143 | 143 | 179 | 183 | 263 | 263 | 250 | 278 | 162 | 162 | 232 | 244 | 449 | 449 |
|  | SRE038 | 143 | 145 | 183 | 183 | 263 | 263 | 162 | 218 | 162 | 162 | 262 | 0 | 0 | 0 |
|  | SRE039 | 143 | 143 | 179 | 181 | 263 | 263 | 222 | 258 | 162 | 162 | 244 | 262 | 449 | 449 |
|  | SRE040 | 143 | 151 | 179 | 179 | 0 | 0 | 222 | 290 | 154 | 154 | 238 | 0 | 443 | 449 |
|  | SRE041 | 143 | 143 | 183 | 183 | 263 | 263 | 162 | 254 | 162 | 162 | 212 | 218 | 449 | 449 |
|  | SRE042 | 143 | 145 | 183 | 183 | 263 | 263 | 262 | 266 | 162 | 162 | 206 | 250 | 383 | 449 |
|  | SRE043 | 143 | 145 | 183 | 183 | 263 | 263 | 230 | 262 | 162 | 162 | 224 | 232 | 449 | 449 |
|  | SRE044 | 141 | 143 | 179 | 183 | 263 | 263 | 226 | 262 | 162 | 162 | 248 | 262 | 443 | 449 |
|  | SRE045 | 143 | 143 | 183 | 183 | 0 | 0 | 162 | 274 | 154 | 162 | 220 | 250 | 449 | 449 |
|  | SRE046 | 143 | 143 | 183 | 183 | 263 | 263 | 0 | 0 | 0 | 0 | 220 | 248 | 449 | 449 |
|  | SRE047 | 143 | 143 | 183 | 183 | 263 | 263 | 258 | 266 | 162 | 162 | 244 | 300 | 449 | 449 |
|  | SRE048 | 143 | 143 | 183 | 183 | 263 | 263 | 226 | 278 | 162 | 162 | 220 | 248 | 449 | 449 |
|  | SRE049 | 145 | 153 | 183 | 183 | 263 | 263 | 278 | 282 | 162 | 162 | 212 | 244 | 443 | 451 |
|  | SRE050 | 141 | 153 | 183 | 183 | 263 | 263 | 222 | 230 | 162 | 162 | 206 | 206 | 449 | 449 |
|  | SRE051 | 143 | 143 | 183 | 183 | 263 | 263 | 254 | 278 | 162 | 162 | 206 | 260 | 443 | 449 |
|  | SRE052 | 143 | 143 | 0 | 0 | 263 | 272 | 262 | 266 | 162 | 162 | 264 | 284 | 443 | 449 |
|  | SRE053 | 143 | 143 | 181 | 183 | 263 | 263 | 258 | 274 | 162 | 162 | 268 | 0 | 451 | 451 |
|  | SRE054 | 143 | 143 | 183 | 183 | 263 | 263 | 162 | 274 | 154 | 162 | 250 | 288 | 449 | 449 |
|  | SRE055 | 0 | 0 | 183 | 183 | 263 | 263 | 254 | 256 | 162 | 162 | 274 | 284 | 449 | 449 |
| COLETON SC | Coleton_SC_PO_012_2006 | 143 | 145 | 183 | 183 | 263 | 263 | 0 | 0 | 162 | 162 | 236 | 276 | 449 | 499 |
|  | Coleton_SC_PO_014_2006 | 143 | 143 | 183 | 183 | 263 | 263 | 0 | 0 | 162 | 162 | 236 | 268 | 449 | 449 |
|  | Coleton_SC_PO_032_2006 | 143 | 145 | 183 | 183 | 263 | 263 | 222 | 234 | 162 | 162 | 240 | 290 | 449 | 449 |
|  | Coleton_SC_PO_033_2006 | 143 | 145 | 183 | 183 | 263 | 263 | 0 | 0 | 162 | 162 | 240 | 262 | 443 | 443 |
|  | Coleton_SC_PO_034_2006 | 143 | 153 | 183 | 183 | 263 | 263 | 0 | 0 | 0 | 0 | 240 | 0 | 0 | 0 |
|  | Coleton_SC_PO_035_2006 | 143 | 143 | 181 | 183 | 263 | 263 | 0 | 0 | 162 | 162 | 244 | 264 | 449 | 449 |
|  | Coleton_SC_PO_036_2006 | 143 | 145 | 183 | 183 | 263 | 278 | 0 | 0 | 162 | 162 | 236 | 252 | 449 | 449 |
|  | Coleton_SC_PO_037_2006 | 143 | 145 | 183 | 183 | 263 | 263 | 0 | 0 | 162 | 162 | 0 | 0 | 392 | 449 |
|  | Coleton_SC_PO_038_2006 | 145 | 145 | 179 | 179 | 263 | 266 | 0 | 0 | 162 | 162 | 256 | 276 | 0 | 0 |
|  | Coleton_SC_PO_039_2006 | 153 | 153 | 183 | 183 | 263 | 263 | 222 | 258 | 162 | 162 | 240 | 252 | 0 | 0 |
|  | Coleton_SC_PO_040_2006 | 143 | 145 | 181 | 183 | 263 | 263 | 0 | 0 | 162 | 162 | 236 | 0 | 449 | 449 |
|  | Coleton_SC_PO_041_2006 | 153 | 153 | 183 | 183 | 263 | 263 | 0 | 0 | 162 | 162 | 264 | 264 | 449 | 449 |
|  | Coleton_SC_PO_042_2006 | 143 | 143 | 183 | 183 | 263 | 263 | 0 | 0 | 162 | 162 | 236 | 240 | 0 | 0 |
|  | Coleton_SC_PO_043_2006 | 143 | 145 | 183 | 183 | 263 | 245 | 0 | 0 | 162 | 162 | 264 | 264 | 449 | 449 |
|  | Coleton_SC_PO_044_2006 | 143 | 153 | 181 | 183 | 0 | 0 | 0 | 0 | 162 | 162 | 236 | 264 | 449 | 457 |
|  | Coleton_SC_PO_045_2006 | 143 | 153 | 183 | 183 | 263 | 263 | 0 | 0 | 162 | 162 | 244 | 252 | 449 | 449 |
|  | Coleton_SC_PO_046_2006 | 143 | 153 | 179 | 183 | 263 | 263 | 0 | 0 | 162 | 162 | 244 | 276 | 0 | 0 |
|  | Coleton_SC_PO_047_2006 | 143 | 145 | 183 | 183 | 263 | 260 | 234 | 246 | 162 | 162 | 236 | 244 | 449 | 449 |
|  | Coleton_SC_PO_048_2006 | 145 | 145 | 183 | 183 | 263 | 266 | 0 | 0 | 162 | 162 | 264 | 264 | 449 | 449 |
|  | Coleton_SC_PO_049_2006 | 143 | 145 | 183 | 183 | 263 | 263 | 258 | 262 | 162 | 162 | 276 | 276 | 443 | 443 |
|  | Coleton_SC_PO_050_2006 | 145 | 153 | 183 | 183 | 263 | 266 | 258 | 262 | 162 | 162 | 256 | 256 | 449 | 449 |
|  | Coleton_SC_PO_051_2006 | 143 | 143 | 183 | 183 | 263 | 266 | 222 | 262 | 162 | 162 | 236 | 236 | 449 | 449 |
|  | Coleton_SC_PO_052_2006 | 145 | 145 | 183 | 183 | 263 | 266 | 234 | 280 | 162 | 162 | 240 | 256 | 449 | 449 |
|  | Coleton_SC_PO_053_2006 | 143 | 145 | 181 | 183 | 263 | 263 | 0 | 0 | 0 | 0 | 0 | 0 | 443 | 443 |
|  | Coleton_SC_PO_054_2006 | 143 | 143 | 181 | 183 | 244 | 263 | 262 | 264 | 162 | 162 | 236 | 236 | 443 | 449 |
|  | Coleton_SC_PO_056_2006 | 0 | 0 | 183 | 183 | 263 | 263 | 162 | 246 | 0 | 0 | 244 | 252 | 449 | 449 |
|  | Coleton_SC_PO_066_2007 | 145 | 145 | 179 | 183 | 263 | 263 | 246 | 246 | 162 | 162 | 220 | 220 | 449 | 449 |
|  | Coleton_SC_PO_067_2007 | 143 | 145 | 179 | 183 | 263 | 266 | 226 | 242 | 162 | 162 | 0 | 0 | 449 | 449 |
|  | Coleton_SC_PO_068_2007 | 145 | 153 | 179 | 179 | 263 | 263 | 238 | 242 | 162 | 162 | 284 | 290 | 449 | 449 |
|  | Coleton_SC_PO_069_2007 | 143 | 143 | 179 | 183 | 263 | 263 | 234 | 238 | 162 | 162 | 244 | 282 | 449 | 449 |
|  | Coleton_SC_PO_070_2007 | 145 | 145 | 179 | 183 | 263 | 266 | 0 | 0 | 162 | 162 | 220 | 222 | 449 | 449 |
|  | Coleton_SC_PO_071_2007 | 143 | 145 | 179 | 183 | 263 | 263 | 238 | 258 | 162 | 162 | 264 | 290 | 449 | 449 |
|  | Coleton_SC_PO_072_2007 | 143 | 143 | 179 | 183 | 263 | 263 | 0 | 0 | 162 | 162 | 224 | 244 | 449 | 449 |
|  | Coleton_SC_PO_073_2007 | 141 | 143 | 183 | 183 | 263 | 266 | 250 | 286 | 162 | 162 | 220 | 224 | 449 | 449 |
|  | Coleton_SC_PO_074_2007 | 145 | 153 | 179 | 183 | 263 | 263 | 238 | 242 | 162 | 162 | 186 | 246 | 449 | 449 |
|  | Coleton_SC_PO_075_2007 | 143 | 145 | 183 | 183 | 263 | 263 | 250 | 250 | 162 | 162 | 232 | 250 | 449 | 469 |
|  | Coleton_SC_PO_076_2007 | 143 | 143 | 183 | 183 | 263 | 263 | 226 | 226 | 162 | 162 | 226 | 226 | 449 | 449 |
|  | Coleton_SC_PO_077_2007 | 143 | 143 | 179 | 183 | 263 | 263 | 238 | 258 | 162 | 162 | 246 | 246 | 449 | 449 |
|  | Coleton_SC_PO_078_2007 | 143 | 145 | 181 | 183 | 263 | 263 | 0 | 0 | 162 | 162 | 220 | 242 | 443 | 449 |
|  | Coleton_SC_PO_079_2007 | 143 | 145 | 181 | 183 | 263 | 263 | 250 | 262 | 162 | 162 | 236 | 244 | 449 | 449 |
|  | Coleton_SC_PO_080_2007 | 145 | 145 | 183 | 183 | 263 | 263 | 0 | 0 | 162 | 162 | 244 | 248 | 449 | 449 |
|  | Coleton_SC_PO_081_2007 | 143 | 143 | 179 | 183 | 263 | 263 | 238 | 258 | 162 | 162 | 246 | 320 | 449 | 451 |
|  | Coleton_SC_PO_082_2007 | 141 | 143 | 181 | 183 | 263 | 263 | 246 | 262 | 162 | 162 | 224 | 244 | 449 | 449 |
|  | Coleton_SC_PO_083_2007 | 0 | 0 | 0 | 0 | 263 | 263 | 0 | 0 | 162 | 162 | 232 | 246 | 449 | 449 |
|  | Coleton_SC_PO_084_2007 | 143 | 143 | 179 | 179 | 263 | 263 | 238 | 242 | 162 | 162 | 246 | 284 | 449 | 449 |
|  | Coleton_SC_b_PO_086_SC_2007 | 143 | 153 | 181 | 183 | 263 | 263 | 234 | 284 | 162 | 162 | 244 | 244 | 443 | 449 |
|  | Coleton_SC_b_PO_089_SC_2007 | 141 | 147 | 183 | 263 | 263 | 263 | 0 | 0 | 162 | 162 | 236 | 264 | 449 | 449 |
|  | Coleton_SC_b_PO_090_SC_2007 | 145 | 153 | 181 | 183 | 263 | 263 | 226 | 262 | 162 | 162 | 264 | 320 | 443 | 449 |
|  | Coleton_SC_b_PO_091_SC_2007 | 143 | 145 | 181 | 183 | 263 | 263 | 246 | 262 | 162 | 162 | 264 | 270 | 443 | 449 |
|  | Coleton_SC_b_PO_092_SC_2007 | 0 | 0 | 0 | 0 | 263 | 263 | 262 | 262 | 162 | 162 | 264 | 244 | 443 | 449 |
|  | Coleton_SC_b_PO_093_SC_2007 | 141 | 145 | 181 | 183 | 263 | 263 | 222 | 272 | 162 | 162 | 244 | 244 | 443 | 449 |
|  | Coleton_SC_b_PO_094_SC_2007 | 143 | 145 | 183 | 183 | 263 | 263 | 246 | 250 | 162 | 162 | 268 | 288 | 0 | 0 |
| FORT BRAGG | Fort_Brag_NT_001_2007 | 141 | 151 | 179 | 179 | 263 | 272 | 242 | 262 | 162 | 162 | 222 | 254 | 384 | 449 |
|  | Fort_Brag_NT_002_2007 | 141 | 151 | 179 | 179 | 263 | 272 | 254 | 258 | 162 | 162 | 222 | 234 | 437 | 449 |
|  | Fort_Brag_NT_003_2007 | 141 | 141 | 179 | 179 | 234 | 272 | 272 | 292 | 162 | 162 | 0 | 0 | 449 | 384 |
|  | Fort_Brag_NT_004_2007 | 151 | 151 | 179 | 179 | 263 | 272 | 258 | 292 | 162 | 162 | 0 | 0 | 384 | 449 |
|  | Fort_Brag_NT_005_2007 | 141 | 151 | 179 | 183 | 263 | 263 | 254 | 292 | 162 | 162 | 234 | 236 | 437 | 449 |
|  | Fort_Brag_NT_006_2007 | 141 | 151 | 179 | 183 | 263 | 263 | 258 | 292 | 154 | 162 | 222 | 234 | 449 | 449 |
|  | Fort_Brag_NT_007_2007 | 141 | 151 | 179 | 179 | 263 | 263 | 258 | 292 | 162 | 166 | 234 | 258 | 449 | 449 |
|  | Fort_Brag_NT_008_2007 | 151 | 107 | 179 | 179 | 263 | 272 | 242 | 292 | 162 | 162 | 0 | 0 | 0 | 0 |
| HARDYVILLE SC | Hardy_SC_PO_058_2006 | 143 | 143 | 183 | 183 | 263 | 263 | 262 | 276 | 154 | 154 | 216 | 260 | 384 | 425 |
|  | Hardy_SC_PO_059_2006 | 0 | 0 | 179 | 181 | 263 | 263 | 264 | 282 | 162 | 162 | 234 | 252 | 449 | 449 |
|  | Hardy_SC_PO_060_2006 | 0 | 0 | 181 | 183 | 263 | 263 | 154 | 250 | 162 | 162 | 224 | 286 | 449 | 449 |
|  | Hardy_SC_PO_061_2006 | 0 | 0 | 181 | 183 | 263 | 263 | 162 | 262 | 162 | 162 | 256 | 260 | 384 | 449 |
|  | Hardy_SC_PO_062_2006 | 145 | 145 | 181 | 183 | 263 | 263 | 154 | 154 | 162 | 162 | 224 | 250 | 449 | 449 |
|  | Hardy_SC_PO_063_2006 | 143 | 143 | 181 | 183 | 263 | 263 | 162 | 246 | 162 | 162 | 246 | 258 | 449 | 449 |
|  | Hardy_SC_PO_064_2006 | 143 | 143 | 181 | 183 | 263 | 263 | 254 | 264 | 162 | 162 | 256 | 256 | 449 | 449 |
|  | Hardy_SC_PO_065_2006 | 143 | 143 | 183 | 183 | 263 | 263 | 258 | 262 | 162 | 162 | 240 | 244 | 449 | 449 |
|  | Hardy_SC_PO_130_2007 | 143 | 153 | 181 | 183 | 263 | 263 | 262 | 282 | 162 | 162 | 216 | 252 | 425 | 449 |
|  | Hardy_SC_PO_131_2007 | 143 | 145 | 181 | 183 | 263 | 263 | 254 | 282 | 162 | 162 | 260 | 260 | 449 | 449 |
|  | Hardy_SC_PO_132_2007 | 143 | 143 | 183 | 183 | 263 | 263 | 162 | 162 | 162 | 162 | 256 | 206 | 449 | 449 |
|  | Hardy_SC_PO_133_2007 | 141 | 143 | 183 | 183 | 263 | 263 | 250 | 276 | 158 | 162 | 248 | 258 | 449 | 449 |
|  | Hardy_SC_PO_134_2007 | 145 | 145 | 181 | 183 | 263 | 263 | 154 | 272 | 162 | 162 | 216 | 252 | 449 | 449 |
|  | Hardy_SC_PO_135_2007 | 143 | 143 | 181 | 183 | 263 | 263 | 254 | 276 | 162 | 162 | 216 | 258 | 449 | 449 |
|  | Hardy_SC_PO_136_2007 | 143 | 145 | 181 | 183 | 263 | 263 | 226 | 262 | 162 | 162 | 242 | 272 | 384 | 449 |
|  | Hardy_SC_PO_137_2007 | 143 | 143 | 181 | 183 | 263 | 263 | 242 | 276 | 162 | 162 | 246 | 246 | 0 | 0 |
|  | Hardy_SC_PO_138_2007 | 143 | 143 | 181 | 183 | 263 | 263 | 222 | 226 | 162 | 162 | 206 | 248 | 449 | 449 |
|  | Hardy_SC_PO_139_2007 | 141 | 143 | 181 | 183 | 263 | 266 | 0 | 0 | 162 | 162 | 250 | 256 | 449 | 449 |
|  | Hardy_SC_PO_140_2007 | 143 | 143 | 181 | 183 | 263 | 263 | 246 | 254 | 162 | 162 | 242 | 242 | 449 | 449 |
|  | Hardy_SC_PO_141_2007 | 145 | 153 | 181 | 183 | 263 | 263 | 276 | 282 | 154 | 162 | 258 | 258 | 449 | 449 |
|  | Hardy_SC_PO_142_2007 | 143 | 153 | 181 | 183 | 263 | 263 | 162 | 222 | 162 | 162 | 244 | 258 | 449 | 449 |
|  | Hardy_SC_PO_143_2007 | 143 | 143 | 181 | 183 | 263 | 263 | 250 | 276 | 158 | 162 | 278 | 280 | 443 | 443 |
|  | Hardy_SC_PO_144_2007 | 143 | 145 | 181 | 183 | 263 | 263 | 262 | 282 | 162 | 162 | 216 | 256 | 443 | 449 |
|  | Hardy_SC_PO_145_2007 | 143 | 145 | 181 | 183 | 263 | 263 | 0 | 0 | 162 | 162 | 246 | 252 | 449 | 449 |
|  | Hardy_SC_PO_146_2007 | 143 | 143 | 181 | 183 | 263 | 263 | 276 | 276 | 162 | 162 | 256 | 260 | 0 | 0 |
| FL HWY 379 | HWY_379_EMC_2209_2006 | 141 | 141 | 179 | 181 | 263 | 263 | 0 | 0 | 162 | 162 | 266 | 266 | 0 | 0 |
|  | HWY_379_EMC_2210_2006 | 143 | 147 | 181 | 181 | 263 | 269 | 272 | 272 | 162 | 162 | 248 | 248 | 449 | 449 |
|  | HWY_379_EMC_2211_2006 | 141 | 143 | 181 | 181 | 263 | 269 | 264 | 264 | 162 | 162 | 0 | 0 | 0 | 0 |
|  | HWY_379_EMC_2212_2006 | 141 | 141 | 181 | 181 | 263 | 263 | 268 | 268 | 162 | 162 | 248 | 248 | 0 | 0 |
|  | HWY_379_EMC_2213_2006 | 147 | 147 | 181 | 181 | 263 | 266 | 260 | 260 | 162 | 162 | 262 | 262 | 0 | 0 |
|  | HWY_379_EMC_2214_2006 | 145 | 145 | 181 | 181 | 263 | 263 | 262 | 268 | 162 | 162 | 222 | 224 | 449 | 449 |
|  | HWY_379_EMC_2215_2006 | 141 | 157 | 181 | 183 | 263 | 269 | 272 | 272 | 162 | 162 | 228 | 252 | 449 | 449 |
|  | HWY_379_EMC_2216_2006 | 141 | 141 | 181 | 181 | 263 | 263 | 258 | 258 | 162 | 162 | 246 | 258 | 449 | 449 |
|  | HWY_379_EMC_2217_2006 | 147 | 157 | 181 | 181 | 263 | 266 | 264 | 264 | 158 | 162 | 254 | 270 | 449 | 449 |
|  | HWY_379_EMC_2218_2006 | 147 | 147 | 181 | 181 | 263 | 263 | 264 | 268 | 162 | 162 | 248 | 250 | 449 | 449 |
|  | HWY_379_EMC_2219_2006 | 147 | 151 | 181 | 181 | 263 | 263 | 0 | 0 | 162 | 162 | 236 | 246 | 413 | 413 |
|  | HWY_379_EMC_2220_2006 | 147 | 147 | 181 | 181 | 263 | 263 | 264 | 264 | 162 | 162 | 248 | 248 | 449 | 449 |
|  | HWY_379_EMC_2221_2006 | 141 | 147 | 181 | 181 | 263 | 263 | 264 | 264 | 162 | 154 | 254 | 258 | 460 | 460 |
|  | HWY_379_EMC_2222_2006 | 141 | 141 | 179 | 181 | 263 | 263 | 250 | 272 | 162 | 162 | 0 | 0 | 449 | 449 |
|  | HWY_379_EMC_2223_2006 | 143 | 157 | 175 | 181 | 263 | 269 | 196 | 260 | 162 | 162 | 250 | 250 | 449 | 449 |
|  | HWY_379_EMC_2369_2006 | 141 | 147 | 181 | 181 | 260 | 278 | 268 | 268 | 162 | 162 | 234 | 236 | 0 | 0 |
|  | HWY_379_EMC_2370_2006 | 141 | 145 | 181 | 181 | 266 | 266 | 250 | 250 | 162 | 162 | 234 | 236 | 449 | 449 |
|  | HWY_379_EMC_2371_2006 | 141 | 143 | 179 | 181 | 263 | 269 | 260 | 264 | 162 | 162 | 228 | 250 | 0 | 0 |
|  | HWY_379_EMC_2372_2006 | 145 | 143 | 181 | 181 | 0 | 0 | 0 | 0 | 162 | 154 | 244 | 250 | 435 | 435 |
|  | HWY_379_EMC_2373_2006 | 147 | 147 | 175 | 181 | 266 | 266 | 0 | 0 | 162 | 162 | 220 | 228 | 0 | 0 |
|  | HWY_379_EMC_2374_2006 | 141 | 141 | 181 | 181 | 266 | 266 | 272 | 272 | 162 | 162 | 232 | 250 | 384 | 449 |
|  | HWY_379_EMC_2375_2006 | 143 | 143 | 181 | 181 | 263 | 269 | 264 | 264 | 162 | 162 | 258 | 0 | 435 | 449 |
|  | HWY_379_EMC_2376_2006 | 141 | 141 | 181 | 181 | 266 | 266 | 252 | 276 | 162 | 162 | 224 | 260 | 449 | 449 |
|  | HWY_379_EMC_2377_2006 | 141 | 141 | 181 | 181 | 263 | 266 | 268 | 272 | 162 | 162 | 244 | 250 | 321 | 321 |
|  | HWY_379_EMC_2378_2006 | 147 | 147 | 181 | 183 | 266 | 266 | 0 | 0 | 162 | 162 | 250 | 258 | 0 | 0 |
|  | HWY_379_EMC_2379_2006 | 141 | 143 | 181 | 181 | 263 | 275 | 0 | 0 | 162 | 162 | 230 | 242 | 384 | 449 |
|  | HWY_379_EMC_2380_2006 | 143 | 143 | 181 | 181 | 263 | 263 | 0 | 0 | 162 | 162 | 226 | 226 | 449 | 449 |
|  | HWY_379_EMC_2381_2006 | 143 | 145 | 181 | 181 | 263 | 266 | 268 | 268 | 162 | 162 | 260 | 260 | 449 | 449 |
|  | HWY_379_EMC_2382_2006 | 141 | 147 | 181 | 181 | 263 | 266 | 280 | 280 | 162 | 162 | 224 | 254 | 0 | 0 |
|  | HWY_379_EMC_2383_2006 | 141 | 143 | 181 | 183 | 263 | 266 | 264 | 264 | 162 | 162 | 238 | 254 | 449 | 449 |
|  | HWY_379_PO_001_2006 | 141 | 141 | 181 | 183 | 0 | 0 | 260 | 260 | 162 | 162 | 224 | 258 | 0 | 0 |
|  | HWY_379_PO_002_2006 | 141 | 141 | 181 | 183 | 263 | 266 | 0 | 0 | 162 | 162 | 216 | 222 | 449 | 449 |
|  | HWY_379_PO_003_2006 | 141 | 143 | 181 | 181 | 266 | 269 | 0 | 0 | 162 | 162 | 230 | 258 | 384 | 489 |
|  | HWY_379_PO_004_2006 | 141 | 143 | 181 | 181 | 263 | 266 | 260 | 260 | 162 | 162 | 0 | 0 | 449 | 449 |
|  | HWY_379_PO_005_2006 | 141 | 143 | 181 | 181 | 263 | 263 | 0 | 0 | 162 | 162 | 254 | 270 | 0 | 0 |
|  | HWY_379_PO_006_2006 | 143 | 145 | 181 | 181 | 263 | 266 | 264 | 272 | 162 | 162 | 240 | 244 | 449 | 449 |
|  | HWY_379_PO_007_2006 | 143 | 143 | 181 | 181 | 263 | 266 | 0 | 0 | 162 | 162 | 226 | 226 | 449 | 449 |
|  | HWY_379_PO_008_2006 | 143 | 141 | 179 | 181 | 263 | 269 | 0 | 0 | 162 | 162 | 242 | 256 | 388 | 449 |
|  | HWY_379_PO_009_2006 | 141 | 147 | 181 | 183 | 242 | 263 | 0 | 0 | 162 | 162 | 0 | 0 | 0 | 0 |
|  | HWY_379_PO_010_2006 | 141 | 147 | 181 | 181 | 263 | 266 | 0 | 0 | 162 | 162 | 226 | 240 | 449 | 449 |
|  | HWY_379_PO_011_2006 | 143 | 147 | 181 | 181 | 263 | 266 | 0 | 0 | 162 | 162 | 238 | 238 | 0 | 0 |
|  | HWY_379_PO_086_2007 | 141 | 141 | 181 | 181 | 263 | 263 | 0 | 0 | 162 | 162 | 244 | 248 | 419 | 489 |
|  | HWY_379_PO_087_2007 | 141 | 141 | 181 | 181 | 263 | 266 | 246 | 276 | 162 | 162 | 248 | 248 | 449 | 449 |
|  | HWY_379_PO_088_2007 | 141 | 141 | 181 | 183 | 263 | 266 | 0 | 0 | 162 | 162 | 248 | 254 | 0 | 0 |
|  | HWY_379_PO_089_2007 | 141 | 147 | 181 | 181 | 263 | 266 | 226 | 252 | 162 | 162 | 224 | 224 | 384 | 449 |
|  | HWY_379_PO_090_2007 | 0 | 0 | 0 | 0 | 263 | 263 | 264 | 264 | 0 | 0 | 0 | 0 | 0 | 0 |
|  | HWY_379_PO_091_2007 | 141 | 141 | 181 | 181 | 263 | 263 | 268 | 282 | 162 | 162 | 226 | 236 | 0 | 0 |
|  | HWY_379_PO_092_2007 | 143 | 145 | 181 | 181 | 263 | 266 | 264 | 264 | 162 | 162 | 226 | 226 | 449 | 449 |
|  | HWY_379_PO_093_2007 | 143 | 151 | 181 | 181 | 0 | 0 | 0 | 0 | 162 | 162 | 266 | 268 | 0 | 0 |
|  | HWY_379_PO_094_2007 | 145 | 147 | 175 | 179 | 263 | 266 | 0 | 0 | 162 | 162 | 216 | 232 | 384 | 384 |
|  | HWY_379_PO_095_2007 | 147 | 147 | 181 | 181 | 263 | 269 | 276 | 306 | 162 | 162 | 216 | 270 | 0 | 0 |
|  | HWY_379_PO_096_2007 | 0 | 0 | 0 | 0 | 263 | 263 | 0 | 0 | 162 | 162 | 236 | 236 | 449 | 449 |
|  | HWY_379_PO_097_2007 | 141 | 145 | 181 | 181 | 263 | 263 | 260 | 268 | 162 | 162 | 250 | 250 | 449 | 449 |
|  | HWY_379_PO_098_2007 | 143 | 147 | 181 | 181 | 263 | 266 | 260 | 260 | 162 | 162 | 246 | 246 | 0 | 0 |
|  | HWY_379_PO_099_2007 | 141 | 145 | 181 | 181 | 263 | 266 | 264 | 264 | 162 | 162 | 226 | 246 | 449 | 449 |
|  | HWY_379_PO_100_2007 | 141 | 147 | 181 | 181 | 263 | 263 | 264 | 268 | 162 | 162 | 258 | 258 | 392 | 449 |
|  | HWY_379_PO_101_2007 | 143 | 143 | 181 | 181 | 263 | 263 | 272 | 280 | 162 | 162 | 242 | 272 | 0 | 0 |
|  | HWY_379_PO_102_2007 | 143 | 143 | 181 | 183 | 266 | 275 | 250 | 260 | 162 | 162 | 224 | 224 | 449 | 489 |
|  | HWY_379_PO_103_2007 | 147 | 147 | 181 | 181 | 263 | 263 | 260 | 260 | 162 | 162 | 230 | 230 | 0 | 0 |
|  | HWY_379_PO_104_2007 | 141 | 141 | 181 | 181 | 263 | 266 | 260 | 276 | 162 | 162 | 224 | 230 | 0 | 0 |
|  | HWY_379_PO_105_2007 | 143 | 143 | 181 | 183 | 263 | 263 | 262 | 264 | 162 | 162 | 248 | 248 | 0 | 0 |
|  | HWY_379_PO_106_2007 | 141 | 141 | 181 | 181 | 263 | 266 | 252 | 272 | 162 | 162 | 226 | 230 | 0 | 0 |
|  | HWY_379_PO_107_2007 | 145 | 147 | 181 | 181 | 266 | 266 | 268 | 268 | 162 | 162 | 244 | 266 | 0 | 0 |
|  | HWY_379_PO_108_2007 | 141 | 147 | 181 | 181 | 263 | 266 | 0 | 0 | 162 | 162 | 256 | 258 | 0 | 0 |
|  | HWY_379_PO_109_2007 | 141 | 141 | 181 | 181 | 263 | 263 | 262 | 276 | 162 | 162 | 224 | 244 | 384 | 435 |
|  | HWY_379_PO_110_2007 | 147 | 147 | 181 | 181 | 263 | 263 | 260 | 264 | 162 | 162 | 244 | 244 | 0 | 0 |
|  | HWY_379_PO_111_2007 | 141 | 141 | 181 | 181 | 263 | 263 | 268 | 272 | 162 | 162 | 228 | 250 | 384 | 449 |
|  | HWY_379_PO_112_2007 | 143 | 143 | 181 | 181 | 263 | 269 | 258 | 258 | 162 | 162 | 244 | 254 | 0 | 0 |
|  | HWY_379_PO_113_2007 | 145 | 147 | 181 | 181 | 263 | 263 | 268 | 268 | 162 | 162 | 226 | 242 | 0 | 0 |
|  | HWY_379_PO_114_2007 | 141 | 143 | 181 | 183 | 263 | 263 | 276 | 276 | 162 | 162 | 230 | 236 | 0 | 0 |
|  | HWY_379_PO_115_2007 | 143 | 143 | 181 | 181 | 263 | 269 | 262 | 268 | 0 | 0 | 224 | 246 | 0 | 0 |
|  | HWY_379_PO_116_2007 | 143 | 143 | 175 | 181 | 266 | 272 | 262 | 262 | 162 | 162 | 226 | 246 | 0 | 0 |
|  | HWY_379_PO_117_2007 | 143 | 143 | 181 | 181 | 263 | 263 | 264 | 264 | 162 | 162 | 222 | 248 | 0 | 0 |
|  | HWY_379_PO_118_2007 | 143 | 143 | 181 | 183 | 266 | 226 | 264 | 264 | 162 | 162 | 230 | 0 | 0 | 0 |
|  | HWY_379_PO_119_2007 | 141 | 147 | 181 | 181 | 263 | 263 | 272 | 272 | 162 | 162 | 236 | 242 | 0 | 0 |
|  | HWY_379_PO_120_2007 | 143 | 147 | 181 | 181 | 263 | 263 | 260 | 268 | 162 | 162 | 0 | 0 | 0 | 0 |
|  | HWY_379_PO_121_2007 | 143 | 143 | 181 | 181 | 272 | 272 | 260 | 260 | 162 | 162 | 238 | 254 | 0 | 0 |
|  | HWY_379_PO_122_2007 | 141 | 147 | 181 | 181 | 263 | 266 | 272 | 272 | 0 | 0 | 244 | 252 | 0 | 0 |
|  | HWY_379_PO_123_2007 | 143 | 145 | 181 | 181 | 263 | 266 | 260 | 260 | 162 | 162 | 222 | 228 | 0 | 0 |
|  | HWY_379_PO_124_2007 | 141 | 145 | 181 | 181 | 263 | 266 | 0 | 0 | 162 | 162 | 274 | 0 | 0 | 0 |
|  | HWY_379_PO_125_2007 | 143 | 145 | 179 | 181 | 263 | 263 | 264 | 242 | 162 | 162 | 238 | 246 | 0 | 0 |
|  | HWY_379_PO_126_2007 | 143 | 147 | 181 | 183 | 263 | 266 | 264 | 264 | 162 | 162 | 254 | 258 | 0 | 0 |
|  | HWY_379_PO_127_2007 | 145 | 145 | 179 | 181 | 263 | 263 | 264 | 264 | 162 | 162 | 0 | 0 | 0 | 0 |
|  | HWY_379_PO_128_2007 | 143 | 147 | 181 | 181 | 263 | 266 | 252 | 264 | 162 | 162 | 240 | 240 | 0 | 0 |
|  | HWY_379_PO_129_2007 | 0 | 0 | 0 | 0 | 263 | 266 | 252 | 252 | 162 | 162 | 0 | 0 | 0 | 0 |
| POND 51 | POND_51_AMH_001_2006 | 141 | 151 | 181 | 181 | 263 | 266 | 258 | 258 | 154 | 162 | 250 | 268 | 449 | 449 |
|  | POND_51_AMH_002_2006 | 143 | 147 | 181 | 181 | 0 | 0 | 0 | 0 | 154 | 162 | 248 | 250 | 384 | 449 |
|  | POND_51_AMH_003_2006 | 143 | 147 | 181 | 181 | 272 | 272 | 0 | 0 | 162 | 162 | 250 | 252 | 384 | 449 |
|  | POND_51_AMH_004_2006 | 141 | 143 | 181 | 181 | 263 | 272 | 272 | 272 | 154 | 162 | 248 | 254 | 384 | 449 |
|  | POND_51_AMH_005_2006 | 0 | 0 | 0 | 0 | 263 | 263 | 272 | 272 | 162 | 162 | 252 | 264 | 449 | 449 |
|  | POND_51_AMH_006_2006 | 143 | 143 | 181 | 181 | 263 | 263 | 258 | 258 | 162 | 162 | 250 | 260 | 328 | 449 |
|  | POND_51_AMH_007_2006 | 143 | 143 | 181 | 181 | 263 | 266 | 0 | 0 | 162 | 162 | 250 | 268 | 449 | 449 |
|  | POND_51_AMH_008_2006 | 143 | 143 | 181 | 181 | 263 | 263 | 0 | 0 | 162 | 162 | 210 | 268 | 449 | 449 |
|  | POND_51_AMH_009_2006 | 0 | 0 | 0 | 0 | 266 | 266 | 272 | 272 | 162 | 162 | 256 | 256 | 449 | 449 |
|  | POND_51_AMH_010_2006 | 0 | 0 | 181 | 183 | 263 | 263 | 272 | 272 | 154 | 162 | 248 | 250 | 384 | 449 |
|  | POND_51_AMH_011_2006 | 143 | 143 | 181 | 181 | 0 | 0 | 272 | 272 | 162 | 162 | 248 | 254 | 449 | 449 |
|  | POND_51_AMH_012_2006 | 141 | 143 | 181 | 181 | 263 | 272 | 0 | 0 | 162 | 162 | 252 | 264 | 384 | 449 |
|  | POND_51_AMH_013_2006 | 143 | 147 | 181 | 181 | 263 | 263 | 238 | 238 | 154 | 162 | 252 | 262 | 384 | 449 |
|  | POND_51_AMH_014_2006 | 141 | 143 | 181 | 181 | 263 | 263 | 272 | 272 | 154 | 162 | 252 | 248 | 449 | 449 |
|  | POND_51_AMH_015_2006 | 141 | 141 | 181 | 183 | 0 | 0 | 0 | 0 | 162 | 162 | 210 | 248 | 449 | 449 |
|  | POND_51_AMH_016_2006 | 141 | 141 | 181 | 181 | 266 | 266 | 212 | 212 | 162 | 162 | 256 | 256 | 384 | 449 |
|  | POND_51_AMH_017_2007 | 143 | 143 | 183 | 183 | 263 | 263 | 272 | 272 | 162 | 162 | 224 | 248 | 449 | 449 |
|  | POND_51_AMH_018_2007 | 143 | 143 | 181 | 183 | 263 | 263 | 0 | 0 | 162 | 162 | 268 | 264 | 386 | 449 |
|  | POND_51_AMH_019_2007 | 143 | 143 | 183 | 183 | 263 | 263 | 0 | 0 | 162 | 162 | 232 | 264 | 449 | 449 |
|  | POND_51_AMH_020_2007 | 143 | 143 | 181 | 183 | 222 | 263 | 264 | 272 | 162 | 162 | 268 | 250 | 449 | 449 |
|  | POND_51_AMH_021_2007 | 143 | 143 | 181 | 181 | 263 | 266 | 272 | 272 | 162 | 162 | 228 | 248 | 443 | 449 |
|  | POND_51_AMH_022_2007 | 141 | 143 | 181 | 181 | 263 | 263 | 258 | 258 | 162 | 162 | 250 | 252 | 449 | 449 |
|  | POND_51_AMH_023_2007 | 143 | 143 | 181 | 181 | 263 | 269 | 234 | 258 | 162 | 162 | 224 | 224 | 449 | 449 |
|  | POND_51_AMH_024_2007 | 141 | 143 | 181 | 181 | 263 | 263 | 0 | 0 | 162 | 162 | 210 | 258 | 449 | 449 |
|  | POND_51_AMH_025_2007 | 141 | 141 | 181 | 183 | 263 | 263 | 180 | 238 | 162 | 162 | 254 | 254 | 449 | 449 |
|  | POND_51_AMH_026_2007 | 143 | 143 | 181 | 183 | 263 | 263 | 276 | 276 | 162 | 162 | 220 | 276 | 449 | 449 |
|  | POND_51_AMH_027_2007 | 0 | 0 | 183 | 183 | 263 | 266 | 290 | 290 | 162 | 162 | 254 | 264 | 449 | 449 |
|  | POND_51_AMH_028_2007 | 143 | 143 | 181 | 181 | 263 | 269 | 0 | 0 | 154 | 162 | 210 | 210 | 449 | 449 |
|  | POND_51_AMH_029_2007 | 141 | 147 | 181 | 181 | 263 | 263 | 264 | 264 | 162 | 162 | 220 | 250 | 449 | 449 |
|  | POND_51_AMH_030_2007 | 141 | 143 | 181 | 181 | 263 | 263 | 272 | 272 | 162 | 162 | 224 | 248 | 384 | 384 |
|  | POND_51_AMH_031_2007 | 143 | 147 | 181 | 181 | 263 | 263 | 258 | 276 | 158 | 162 | 226 | 256 | 449 | 449 |
|  | POND_51_AMH_032_2007 | 143 | 147 | 181 | 181 | 263 | 263 | 234 | 286 | 162 | 162 | 250 | 254 | 449 | 449 |
|  | POND_51_AMH_034_2007 | 141 | 143 | 181 | 181 | 263 | 266 | 294 | 294 | 162 | 162 | 262 | 266 | 449 | 449 |
|  | POND_51_AMH_035_2007 | 143 | 143 | 181 | 183 | 263 | 272 | 276 | 276 | 162 | 154 | 210 | 254 | 443 | 449 |
|  | POND_51_AMH_036_2007 | 143 | 153 | 183 | 183 | 263 | 266 | 284 | 294 | 154 | 162 | 210 | 272 | 449 | 449 |
|  | POND_51_AMH_037_2007 | 143 | 153 | 183 | 183 | 263 | 263 | 256 | 256 | 162 | 162 | 220 | 224 | 328 | 449 |
|  | POND_51_AMH_038_2007 | 145 | 153 | 183 | 183 | 263 | 266 | 258 | 258 | 162 | 162 | 230 | 278 | 449 | 449 |

Table B. Information on species population locations, number of individuals in populations and year collected.

| *Species* | *Population* | *Latitude* | *Longitude* | *N* | *Year Collected* |
| --- | --- | --- | --- | --- | --- |
| *Notophthalmus perstriatus* | ONF 1 | 29.383 | -81.7956 | 27 | 2008 |
|  | ONF 2 | 29.416111 | -81.761111 | 31 | 1997-2000 |
|  | ONF 3 | 29.060833 | -81.803389 | 44 | 2009 |
|  | ONF 4 | 29.055833 | -81.560389 | 23 | 2009 |
|  | Berry | 29.6877 | -82.006831 | 13 | 2008 |
|  | FD | 29.680753 | -81.265833 | 34 | 2002, 2009-2010 |
|  | GSF | 29.53475 | -82.597861 | 23 | 2010 |
|  | RSR | 28.775278 | -81.455833 | 18 | 1998, 2010 |
|  | FLSHNA | 32.578194 | -84.269353 | 10 | 2009 |
|  | JSF | 30.001717 | -81.015419 | 14 | 1999, 2009 |
|  | OR1 | 29.69139 | -82.00306 | 98 | 1998, 2008 |
|  | OR2 | 29.7222 | -81.01651 | 29 | 1997-1998, 2008 |
|  | OR3 | 29.01158 | -81.01599 | 17 | 1997, 1999 |
|  | FSMI | n/a | n/a | 29 | 1997 |
|  | CB | 29.015947 | -81.015647 | 17 | 1997, 2009 |
|  | JOH | 29.005383 | -81.012447 | 6 | 1996 |
|  | LA | 29.007275 | -81.012928 | 8 | 1997 |
|  | JP | n/a | n/a | 1 | 2009 |
|  | TE | 29.007278 | -81.012714 | 10 | 2000 |
|  | ANF | 30.339556 | -84.320467 | 8 | 2009 |
|  | JJ | 31.00435 | -84.008817 | 10 | 1998 |
| *Hyla Squirella* | AST | 29.1605 | -81.5535 | 20 | 2010 |
|  | CHAR | 26.9317 | -81.7607 | 1 | 2010 |
|  | CUT | 29.5505 | -83.1829 | 27 | 2010 |
|  | DISS | 29.2771 | -81.3343 | 33 | 2010 |
|  | EAPP | 30.0282 | -84.9879 | 35 | 2010 |
|  | GRAS | 29.0147 | -82.3232 | 10 | 2010 |
|  | GULF | 28.539 | -82.6171 | 37 | 2010 |
|  | HIKE | 30.3461 | -83.3394 | 33 | 2010 |
|  | LAZY | 28.6266 | -81.8882 | 0 | 2010 |
|  | OCK | 29.5376 | -81.778 | 16 | 2010 |
|  | OST | 28.8461 | -81.0936 | 22 | 2010 |
|  | PALM | 27.9213 | -80.5515 | 19 | 2010 |
|  | PEN | 30.3196 | -87.2634 | 36 | 2010 |
|  | PICK | n/a | n/a | 36 | 2010 |
|  | PINE | 30.0503 | -81.3978 | 31 | 2010 |
|  | SAND | 30.2744 | -82.2845 | 32 | 2010 |
|  | SPAR | 29.3811 | -82.042 | 46 | 2010 |
|  | SR2 | 30.3849 | -86.3761 | 41 | 2010 |
|  | STAR | 29.9711 | -82.2559 | 48 | 2010 |
|  | WAPP | 30.1358 | -85.3702 | 41 | 2010 |
|  | WAY | 31.2089 | -82.4494 | 16 | 2010 |
| *Pseudacris ornata* | SRE A | 33.157467 | -81.67625 | 15 | 2006-2009 |
|  | SRE B | 33.160517 | -81.690650 | 1 | 2006-2009 |
|  | SRE C | 33.289383 | -81.48165 | 32 | 2006-2009 |
|  | SRE D | 33.31805 | -81.47685 | 20 | 2006-2009 |
|  | JEN C | 30.160083 | -81.889267 | 9 | 2006-2009 |
|  | JEN I | 30.15195 | -81.8787 | 10 | 2006-2009 |
|  | JEN J | 30.156933 | -81.884617 | 8 | 2006-2009 |
|  | SR A | n/a | n/a | 2 | 2006-2009 |
|  | TEL | 31.861444 | -82.812075 | 6 | 2006-2009 |
|  | AL HWY 165 | 32.0371 | -85.0839 | 5 | 2006-2009 |
|  | Coleton SC | 33.0546 | -80.4859 | 45 | 2006-2009 |
|  | Coleton SC b | 33.0422 | -80.4277 | 7 | 2006-2009 |
|  | Gulf | n/a | n/a | 1 | 2006-2009 |
|  | Hardyville SC | 32.4371 | -81.0042 | 25 | 2006-2009 |
|  | Hwy 379 | 30.0861 | -85.0404 | 85 | 2006-2009 |
|  | Pond 51 | 31.2498 | -84.4947 | 37 | 2006-2009 |
|  | Fort Bragg | 35.135846 | -79.041152 | 8 | 2006-2009 |

Table C. *Bd* and *Rv* infection prevalence with 95% confidence intervals and average *Bd* intensity with standard error for sampled populations of *H. squirella* and *P. ornata*.

| *Population* | *No. Bd infected/No. sampled* | *Bd prevalence (95% CI)* | | *Bd intensity* | *Log(Bd intensity (± SE)* | | *No. Rv infected/No. sampled* | *Rv prevalence (95% CI)* | *Rv intensity*  *(± SE)* |  |  |  |  |  |
| --- | --- | --- | --- | --- | --- | --- | --- | --- | --- | --- | --- | --- | --- | --- |
| *Hyla squirella* | | |  | | |  | |  | | |  | *Hyla squirella* |  |  |
| PICK | 1/36 | 0.03 (0.0007 – 0.15) | | 20687.9 | 9.94 (NA) | | 0/36 | 0 (0- 0.0973) | 0 |  |  |  |  |  |
| AST | 0/20 | 0 ( 0 – 0.17) | | 0 |  | | 0/20 | 0 ( 0 – 0.17) | 0 |  |  |  |  |  |
| CHAR | 0/1 | 0 ( 0 – 0.98) | | 0 |  | | 0/1 | 0 ( 0 – 0.98) | 0 |  |  |  |  |  |
| CUT | 0/27 | 0 ( 0 – 0.13) | | 0 |  | | 1/27 | 0.30 ( 0.0009 – 0.19) | 66.3 (NA) |  |  |  |  |  |
| DISS | 0/33 | 0 ( 0 – 0.11) | | 0 |  | | 0/33 | 0 ( 0 – 0.11) | 0 |  |  |  |  |  |
| EAPP | 0/35 | 0 ( 0 – 0.10) | | 0 |  | | 0/35 | 0 ( 0 – 0.10) | 0 |  |  |  |  |  |
| GRAS | 0/10 | 0 ( 0 – 0.31) | | 0 |  | | 0/10 | 0 ( 0 – 0.31) | 0 |  |  |  |  |  |
| GULF | 0/37 | 0 ( 0 – 0.09) | | 0 |  | | 0/37 | 0 ( 0 – 0.09) | 0 |  |  |  |  |  |
| HIKE | 0/33 | 0 ( 0 – 0.11) | | 0 |  | | 0/33 | 0 ( 0 – 0.11) | 0 |  |  |  |  |  |
| OCK | 0/16 | 0 ( 0 – 0.21) | | 0 |  | | 0/16 | 0 ( 0 – 0.21) | 0 |  |  |  |  |  |
| OST | 0/22 | 0 ( 0 – 0.15) | | 0 |  | | 0/22 | 0 ( 0 – 0.15) | 0 |  |  |  |  |  |
| PALM | 0/19 | 0 ( 0 – 0.18) | | 0 |  | | 0/19 | 0 ( 0 – 0.18) | 0 |  |  |  |  |  |
| PEN | 0/36 | 0 ( 0 – 0.10) | | 0 |  | | 0/36 | 0 ( 0 – 0.10) | 0 |  |  |  |  |  |
| PINE | 0/31 | 0 ( 0 – 0.11) | | 0 |  | | 0/31 | 0 ( 0 – 0.11) | 0 |  |  |  |  |  |
| SAND | 0/32 | 0 ( 0 – 0.11) | | 0 |  | | 0/32 | 0 ( 0 – 0.11) | 0 |  |  |  |  |  |
| SPAR | 0/46 | 0 ( 0 – 0.08) | | 0 |  | | 0/46 | 0 ( 0 – 0.08) | 0 |  |  |  |  |  |
| SR2 | 0/41 | 0 ( 0 – 0.09) | | 0 |  | | 0/41 | 0 ( 0 – 0.09) | 0 |  |  |  |  |  |
| STAR | 0/48 | 0 ( 0 – 0.07) | | 0 |  | | 0/48 | 0 ( 0 – 0.07) | 0 |  |  |  |  |  |
| WAPP | 0/41 | 0 ( 0 – 0.09) | | 0 |  | | 0/41 | 0 ( 0 – 0.09) | 0 |  |  |  |  |  |
| WAY | 0/16 | 0 ( 0 – 0.21) | | 0 |  | | 0/16 | 0 ( 0 – 0.21) | 0 |  |  |  |  |  |
| *Pseudacris ornata* | | |  | | |  | |  | | |  | *Pseudacris ornata* |  |  |
| SRE A | 7/15 | 0.47 (0.21 – 0.73) | | 1237326.8 | 14.03 (1.36) | | 0/15 | 0 (0-0.218) | 0 |  |  |  |  |  |
| SRE B | 1/1 | 1 (0.03 – 1) | | 5731.7 | 8.65(NA) | | 0/1 | 0 (0-0.975) | 0 |  |  |  |  |  |
| SRE C | 16/32 | 0.5 (0.32 – 0.68) | | 360400.5 | 12.79(0.67) | | 0/32 | 0 (0-0.11) | 0 |  |  |  |  |  |
| SRE D | 7/20 | 0.35 (0.15 – 0.59) | | 786777.0 | 13.58(1.26) | | 0/20 | 0 (0-.17) | 0 |  |  |  |  |  |
| JEN C | 0/9 | 0 ( 0 – 0.34) | | 0 |  | | 0/9 | 0 ( 0 – 0.34) | 0 |  |  |  |  |  |
| JEN I | 0/10 | 0 ( 0 – 0.31) | | 0 |  | | 0/10 | 0 ( 0 – 0.31) | 0 |  |  |  |  |  |
| JEN J | 0/8 | 0 ( 0 – 0.37) | | 0 |  | | 0/8 | 0 ( 0 – 0.37) | 0 |  |  |  |  |  |
| TEL | 6/6 | 1 (0.54 – 1) | | 68099.1 | 11.13(0.26) | | 0/6 | 0 (0-0.46) | 0 |  |  |  |  |  |
| AL HWY 165 | 3/5 | 0.6 (0.15 – 0.95) | | 226.1 | 5.42(0.53) | | 0/5 | 0 (0-0.52) | 0 |  |  |  |  |  |
| Coleton SC | 22/45 | 0.49 (0.34 – 0.64) | | 1818694.7 | 14.41(0.82) | | 0/45 | 0 (0-0.078) | 0 |  |  |  |  |  |
| Coleton SC b | 0/7 | 0 ( 0 – 0.41) | | 0 |  | | 0/7 | 0 ( 0 – 0.41) | 0 |  |  |  |  |  |
| Gulf | 0/1 | 0 ( 0 – 0.98) | | 0 |  | | 0/1 | 0 ( 0 – 0.98) | 0 |  |  |  |  |  |
| Hardyville SC | 6/25 | 0.24 (0.09 – 0.45) | | 500392.9 | 13.12(1.28) | | 0/25 | 0 (0-0.137) | 0 |  |  |  |  |  |
| Hwy 379 | 26/85 | 0.31 (0.21 – 0.42) | | 561268.8 | 13.24(0.60) | | 0/85 | 0 (0-0.0424) | 0 |  |  |  |  |  |
| Pond 51 | 0/37 | 0 ( 0 – 0.09) | | 0 |  | | 0/37 | 0 ( 0 – 0.09) | 0 |  |  |  |  |  |
| Savannah R., Ellentor | 0/2 | 0 ( 0 – 0.84) | | 0 |  | | 0/2 | 0 ( 0 – 0.84) | 0 |  |  |  |  |  |
| Savannah R., Mona | 0/5 | 0 ( 0 – 0.52) | | 0 |  | | 0/5 | 0 ( 0 – 0.52) | 0 |  |  |  |  |  |
| Fort Brag NT | 7/8 | .88 (0.47 – .99) | | 17982320.2 | 16.70(0.70) | | 0/8 | 0 (0-0.369) | 0 |  |  |  |  |  |
|  |  |  | |  |  | |  |  |  |  |  |  |  |  |

Table D. Summary estimates of the best fit model for *Bd* prevalence.

|  | *Estimate* | *Std. Error* | *z value* | *p value* |
| --- | --- | --- | --- | --- |
| (Intercept) | -16.60610 | 4.02219 | -4.129 | 0.0000365 |
| PC1 | 0.18931 | 0.03626 | 5.221 | 0.0000002 |
| PC2 | -0.54575 | 0.15885 | -3.436 | 0.000591 |
| Avg.HE | 30.91722 | 7.77433 | 3.977 | 0.0000698 |

Table E. Summary estimates of the best fit model for *Bd* intensity.

|  | Estimate | Std. Error | t-value | p-value |
| --- | --- | --- | --- | --- |
| (Intercept) | 8.1948 | 1.4861 | 5.514 | 0.000182 |
| PC1 | 0.7778 | 0.2559 | 3.039 | 0.011271 |


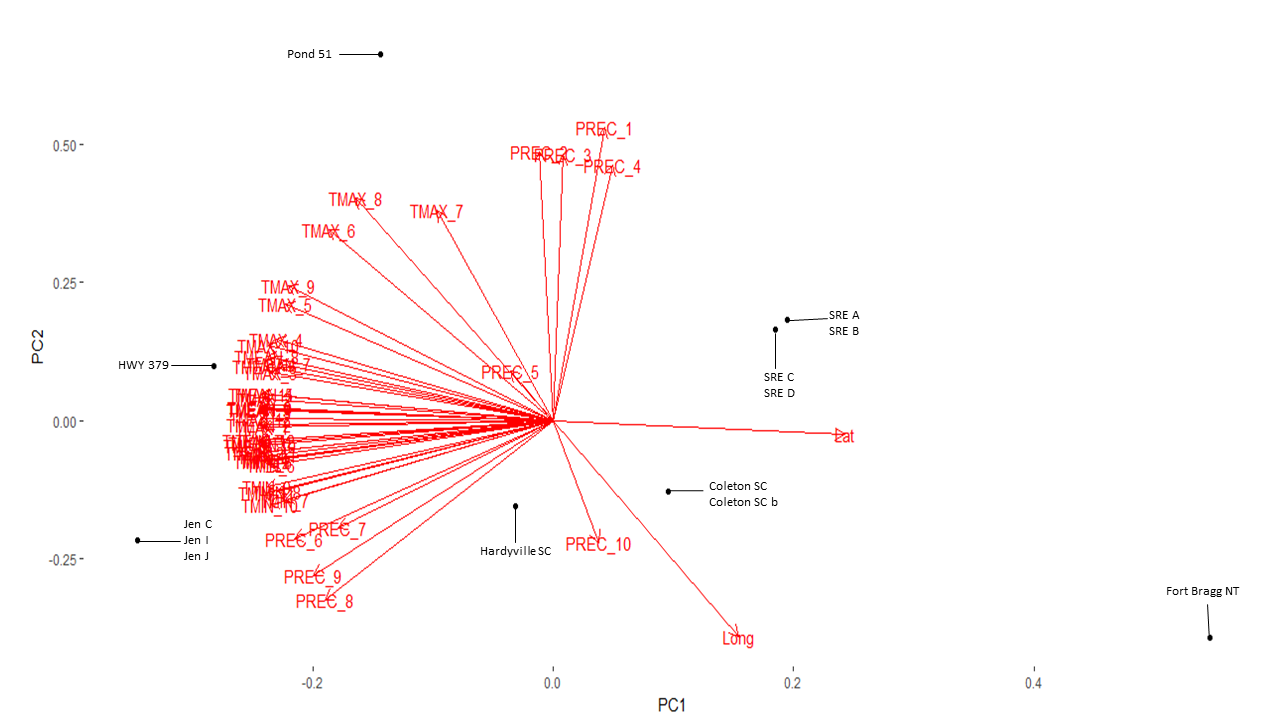


Figure A. Principle Component Analysis (PCA) of environmental factors (maximum and minimum temperatures, average precipitation, and mean temperature per population sites; 36 temperature variables and 12 precipitation variables from online WorldClim database) and location (latitude and longitude).


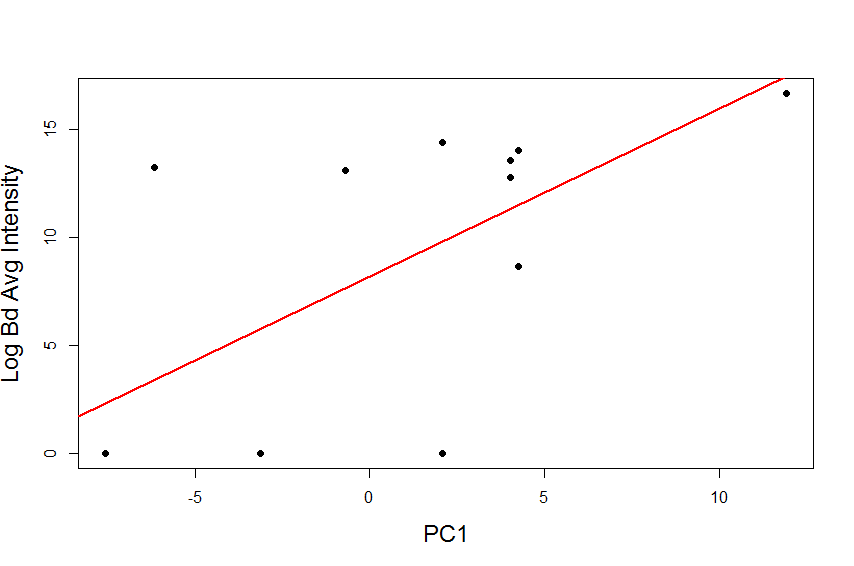


Figure B. Linear relationship between natural log-transformed average *Bd* infection intensity and PC1.

Data A

R Code

#####Standard error########

#function to caluclulate standard error

tapply(intensity$Average.Bd.Load,intensity$Pop.ID, se)

se <- function(x) sqrt(var(x)/length(x))

tapply(intensity$Average.Bd.Load,intensity$Pop.ID, se)

######BinomCIs###########

#make sure you have installed the package binom first!

library(binom)

binom.confint(x=a, n=b)

# so for example if you have 20 out of 50 individuals testing positive

# then x=20 and n=50

#the results generate eight different CI calculations

#clapper-Person is listed as "exact" in the output

R Code For PCA/GLMS:

####P ornata Bd analysis###

data<- read.delim("Bd Master List- all species updated_final3_wo_na.txt", na.strings="n/a", header=T)##change what data you are reading##

datapca<-data[11:58] ##limiting data##

row.names(datapca)<-data$Sub.Population

library(bbmle)

library(car)

library(vegan)

library(ggplot2)

library(ggfortify)

##running PCA##

prcomp(datapca, scale=T)

enviropca<-prcomp(datapca, scale=T)

summary(enviropca)

##Teasing apart the PCA##

autoplot(enviropca, loadings=T, loadings.label=T)+ theme(panel.background = element_blank())

biplot(enviropca, cex.axis=2, cex.lab=3, xlab="PC1 (76%)", ylab="PC2 (13%)", xlim=c(-.5, .6))

#### From this graph we can see that PC1 is inversely related to all temp variables

#### and a few prep varibles. PC2 is primarily associated directely with winterish precip and

#### inversely with Prec_10

load<-enviropca$rotation ##no Standard dev., just rotation data (loadings)##

axes<-predict(enviropca, newdata=data) ##put data in format for regression##

dat<-cbind(data,axes) ##adds column bind; just adds PC variables to my file##

environintens<-lm(log(Bd.Average.intensity+1)~PC1+PC2+Avg.HE+AR, data=dat) ##linear regression, have to add 1 to intensity b/c zeros##

summary(environintens)

##if you forget the names of headers##

names(dat)

##Linear(for now) regression##

prev<-lm(Bd.Prevalence~PC1, data=dat)

prevgen<-lm(Bd.Prevalence~Avg.HE+AR, data=dat)

summary(prev)

#####Looking at potential iteraction terms

modelINT1a<-lm(log(Bd.Average.intensity+1)~PC1*PC2, data=dat)

modelINT2a<-lm(log(Bd.Average.intensity+1)~PC1*Avg.HE, data=dat)

modelINT3a<-lm(log(Bd.Average.intensity+1)~PC1*AR, data=dat)

modelINT4a<-lm(log(Bd.Average.intensity+1)~PC2*AR, data=dat)

modelINT5a<-lm(log(Bd.Average.intensity+1)~PC2*Avg.HE, data=dat)

modelINT6a<-lm(log(Bd.Average.intensity+1)~AR*Avg.HE, data=dat)

#Vifs for interactive models

vif(modelINT1a)

vif(modelINT2a)

vif(modelINT3a)

vif(modelINT4a)

vif(modelINT5a)

vif(modelINT6a)

##Models for Intensity to run through AICs##

model1a<-lm(log(Bd.Average.intensity+1)~PC1+PC2, data=dat)

model2a<-lm(log(Bd.Average.intensity+1)~Avg.HE+AR, data=dat)

model3a<-lm(log(Bd.Average.intensity+1)~PC1, data=dat)

model4a<-lm(log(Bd.Average.intensity+1)~PC2, data=dat)

model5a<-lm(log(Bd.Average.intensity+1)~Avg.HE, data=dat)

model6a<-lm(log(Bd.Average.intensity+1)~AR, data=dat)

model7a<-lm(log(Bd.Average.intensity+1)~PC1+PC2+Avg.HE, data=dat)

model8a<-lm(log(Bd.Average.intensity+1)~PC1+PC2+AR, data=dat)

model9a<-lm(log(Bd.Average.intensity+1)~PC1+Avg.HE, data=dat)

model10a<-lm(log(Bd.Average.intensity+1)~PC1+AR, data=dat)

model11a<-lm(log(Bd.Average.intensity+1)~PC2+AR, data=dat)

model12a<-lm(log(Bd.Average.intensity+1)~PC2+Avg.HE, data=dat)

model13a<-lm(log(Bd.Average.intensity+1)~PC1+Avg.HE+AR, data=dat)

model14a<-lm(log(Bd.Average.intensity+1)~PC2+Avg.HE+AR, data=dat)

model15a<-lm(log(Bd.Average.intensity+1)~PC1+PC2+Avg.HE+AR, data=dat)

model16a<-lm(log(Bd.Average.intensity+1)~1, data=dat)

AICctab(model1a, model2a, model3a, model4a, model5a, model6a, model7a, model8a, model9a, model10a, model11a, model12a, model13a, model14a, model15a, model16a, weights=TRUE, base=TRUE)

summary(model3a)

##Plotting Intesnity models##

plot(dat$PC1,log(dat$Bd.Average.intensity+1), xlab="PC1", ylab="Log Bd Avg Intensity", pch=16, cex.lab=1.5)

abline(model3a, col="red", lwd=2)

#Looking at potenial interactions in prevalence

modelint1b<-glm(Bd.Prevalence~PC1*PC2, data=dat, family=binomial, weight=Number)

modelint2b<-glm(Bd.Prevalence~PC1*Avg.HE, data=dat, family=binomial, weight=Number)

modelint3b<-glm(Bd.Prevalence~PC1*AR, data=dat, family=binomial, weight=Number)

modelint4b<-glm(Bd.Prevalence~PC2*Avg.HE, data=dat, family=binomial, weight=Number)

modelint5b<-glm(Bd.Prevalence~PC2*AR, data=dat, family=binomial, weight=Number)

modelint6b<-glm(Bd.Prevalence~Avg.HE*AR, data=dat, family=binomial, weight=Number)

#looking at potenial interactions in prevalence (vifs)

vif(modelint1b)

vif(modelint2b)

vif(modelint3b)

vif(modelint4b)

vif(modelint5b)

vif(modelint6b)

##Models for Prevalence##

model1b<-glm(Bd.Prevalence~PC1+PC2, data=dat, family=binomial, weight=Number)

model2b<-glm(Bd.Prevalence~Avg.HE+AR, data=dat, family=binomial, weight=Number)

model3b<-glm(Bd.Prevalence~PC1, data=dat, family=binomial, weight=Number)

model4b<-glm(Bd.Prevalence~PC2, data=dat, family=binomial, weight=Number)

model5b<-glm(Bd.Prevalence~Avg.HE, data=dat, family=binomial, weight=Number)

model6b<-glm(Bd.Prevalence~AR, data=dat, family=binomial, weight=Number)

model7b<-glm(Bd.Prevalence~PC1+PC2+Avg.HE, data=dat, family=binomial, weight=Number)

model8b<-glm(Bd.Prevalence~PC1+PC2+AR, data=dat, family=binomial, weight=Number)

model9b<-glm(Bd.Prevalence~PC1+Avg.HE, data=dat, family=binomial, weight=Number)

model10b<-glm(Bd.Prevalence~PC1+AR, data=dat, family=binomial, weight=Number)

model11b<-glm(Bd.Prevalence~PC2+AR, data=dat, family=binomial, weight=Number)

model12b<-glm(Bd.Prevalence~PC2+Avg.HE, data=dat, family=binomial, weight=Number)

model13b<-glm(Bd.Prevalence~PC1+Avg.HE+AR, data=dat, family=binomial, weight=Number)

model14b<-glm(Bd.Prevalence~PC2+Avg.HE+AR, data=dat, family=binomial, weight=Number)

model15b<-glm(Bd.Prevalence~PC1+PC2+Avg.HE+AR, data=dat, family=binomial, weight=Number)

model16b<-glm(Bd.Prevalence~1, data=dat, family=binomial, weight=Number)

AICctab(model1b, model2b, model3b, model4b, model5b, model6b, model7b, model8b, model9b, model10b, model11b, model12b, model13b, model14b, model15b,model16b, weights=TRUE, base=TRUE)

summary(model7b)

##Plotting Prevalence models##

par( cex.lab=3.5, cex.axis=2.2, mai=c(1,1,1,.3), mfrow=c(1,3))

plot(dat$PC1, data$Bd.Prevalence, ylab="", xlab="", pch=16)

title(ylab="Prevalence", line=4.5, xlab="PC1", at=4.5)

vsize<-seq(-8,12,2)

predPC1<-predict(model7b, type="response", list(PC1=vsize, PC2=rep(mean(dat$PC2), length(vsize)), Avg.HE=rep(mean(dat$Avg.HE), length(vsize))))

lines(vsize,predPC1, col="red", lwd=2)

mtext(at=-7,line=-2.5,"A", cex=2.4)

plot(dat$PC2, data$Bd.Prevalence,ylab="" , xlab="", pch=16)

title(ylab="Prevalence", line=4.5, xlab="PC2", at=4.5)

vsize<-seq(-4,7,2)

predPC2<-predict(model7b, type="response", list(PC2=vsize, PC1=rep(mean(dat$PC1), length(vsize)), Avg.HE=rep(mean(dat$Avg.HE), length(vsize))))

lines(vsize,predPC2, col="red", lwd=2)

mtext(at=-2.5,line=-2.5,"B", cex=2.4)

plot(dat$Avg.HE, data$Bd.Prevalence, ylab="", xlab="", pch=16)

title(ylab="Prevalence", line=4.5, xlab="Avg.HE")

vsize<-seq(.45,.6,.02)

predAvg.HE<-predict(model7b, type="response", list(Avg.HE=vsize, PC2=rep(mean(dat$PC2), length(vsize)), PC1=rep(mean(dat$PC1), length(vsize))))

lines(vsize,predAvg.HE, col="red", lwd=2)

mtext(at=.48,line=-2.5,"C", cex=2.4)

#####

a<-as.matrix(data[1:20,6:7])

rownames(a)<-data$Sub.Population[1:20]

fisher.test(a, simulate.p.value=T)

fisher.multcomp(a)

b<-as.matrix(data[20:40,6:7])

rownames(b)<-data$Sub.Population[20:40]

fisher.test(b, simulate.p.value=T)

c<-matrix(c(100,1, 257, 579), ncol=2)

fisher.test(c, simulate.p.value=T)
